# Adaptive rewiring in nonuniform coupled oscillators

**DOI:** 10.1162/netn_a_00211

**Published:** 2022-02-01

**Authors:** MohamamdHossein Manuel Haqiqatkhah, Cees van Leeuwen

**Affiliations:** Brain and Cognition Research Unit, KU Leuven, Leuven, Belgium; Department of Methodology and Statistics, Utrecht University, Utrecht, The Netherlands; Center for Cognitive Science, TU Kaiserslautern, Kaiserslautern, Germany

**Keywords:** Evolving neural networks, Neural oscillators, Dynamical systems, Complexity

## Abstract

Structural plasticity of the brain can be represented in a highly simplified form as adaptive rewiring, the relay of connections according to the spontaneous dynamic synchronization in network activity. Adaptive rewiring, over time, leads from initial random networks to brain-like complex networks, that is, networks with modular small-world structures and a rich-club effect. Adaptive rewiring has only been studied, however, in networks of identical oscillators with uniform or random coupling strengths. To implement information-processing functions (e.g., stimulus selection or memory storage), it is necessary to consider symmetry-breaking perturbations of oscillator amplitudes and coupling strengths. We studied whether nonuniformities in amplitude or connection strength could operate in tandem with adaptive rewiring. Throughout network evolution, either amplitude or connection strength of a subset of oscillators was kept different from the rest. In these extreme conditions, subsets might become isolated from the rest of the network or otherwise interfere with the development of network complexity. However, whereas these subsets form distinctive structural and functional communities, they generally maintain connectivity with the rest of the network and allow the development of network complexity. Pathological development was observed only in a small proportion of the models. These results suggest that adaptive rewiring can robustly operate alongside information processing in biological and artificial neural networks.

## Author Summary

Dynamic synchronization in coupled oscillators has been studied extensively. Previously, it has been shown how these dynamics can adaptively rewire a random network structure into a complex, brain-like one. For biological and artificial networks to benefit from this dynamic self-organization, these networks must have input and memory facilities. Both functions involve breaking the symmetry of uniform network oscillators and network connectivity strength. We show that adaptive rewiring is generally robust against such perturbations. Notably, we show—via novel methods of comparing network structures—that local symmetry-breaking perturbations can develop discernible anatomical and functional connectivity structures at the global level. Our research qualifies adaptive rewiring as a potential tool for optimizing connectivity in biological and artificial neural networks.

## INTRODUCTION

The anatomical connectivity of the brain network is shaped dynamically through **structural plasticity** (Butz, Wörgötter, & van Ooyen, 2009). A variety of structural plasticity mechanisms serve the adaptive role of accommodating the functional connectivity, the mutual statistical dependencies between the network components (Avena-Koenigsberger, Misic, & Sporns, 2018; Rubinov, Sporns, van Leeuwen, & Breakspear, 2009). A common principle underlying these mechanisms has become known as *adaptive rewiring* (Gong & van Leeuwen, 2003, 2004; Papadopoulos, Kim, Kurths, & Bassett, 2017). Adaptive rewiring implements the Hebbian principle of “what fires together, wires together” at the level of network dynamics (Bi & Poo, 2001; Hebb, 1949).

In networks of which the units may represent spiking model neurons (Kwok, Jurica, Raffone, & van Leeuwen, 2007) or **neural mass oscillators** (Rubinov et al., 2009), adaptive rewiring involves establishing connections between dynamically synchronized units at the expense of currently non-synchronized ones. Over time, adaptively rewiring networks dynamically evolve into complex architectures, showing the characteristics of small-worldness, modularity, and the rich-club effect (Gong & van Leeuwen, 2003, 2004; Hellrigel, Jarman, & van Leeuwen, 2019; Rubinov et al., 2009). Since small-worldness (Sporns & Zwi, 2004), modularity (Meunier, Lambiotte, & Bullmore, 2010), and the rich-club effect (van den Heuvel & Sporns, 2011) are characteristics of large-scale brain connectivity, adaptive rewiring could represent a driving force for the dynamic evolution of brain connectivity structure.

Adaptive rewiring models typically are highly simplified models, of which the units are identical nonlinear oscillators with uniform connection weights and coupling strengths (Rubinov et al., 2009). These simplifications severely reduce the functionality of such models. To implement information processes in these networks, it is vital to enable nonidentical oscillators and nonuniform connection strength. However, the question should be asked whether the properties of adaptive rewiring are robust to such nonuniformities. We therefore aimed to explore adaptive rewiring of coupled oscillators with nonuniform amplitude and coupling strengths and compare their evolution with that of uniform networks. The results will be crucial for the utility of adaptive rewiring in biological and artificial neural networks with information-processing functions such as pattern recognition and learning.

### Adaptive Rewiring in Coupled Logistic Maps

Most adaptive rewiring studies have represented network activity by a model known as coupled logistic maps (Kaneko, 1992). This choice was reached based on a succession of abstractions. Mass activity was described by **chaotic attractor** dynamics, shown in Figure S1A of the Supporting Information (Breakspear, Terry, & Friston, 2003). Further simplification was obtained via the **Poincaré section**, yielding the mapping in Figure S1B. This mapping, in turn, can approximately be described, minus the noise, by a logistic map (Figure S1C). Thus, the logistic map is the most straightforward possible abstract representation of neural mass activity (Rubinov et al., 2009). Note that in coupled map networks, the couplings provide noise perturbations to the oscillators, yielding mappings again more similar to Figure S1B.

The version of the logistic map used in our study is shown in Equation 1, in which *x* is a continuous variable in the range [−1, 1] that is updated in discrete time *t*, and *α* is the *amplitude*. Logistic maps are known to exhibit universal dynamical properties (Feigenbaum, 1978). For certain regimes of *α*, the behavior of the logistic map converges to one or more **limit-cycle attractors**, but otherwise, it exhibits chaotic behavior. In these regimes, logistic maps produce deterministic bounded time series that, indeed, qualitatively resemble the oscillations of neural mass activity (see Figure S1).

$$x_{t+1}=1-\alpha x_{t}^{2}.$$ (1)

Because of the universal dynamics of logistic maps, networks of such simple maps may capture generic properties of interacting nonlinear systems (Kaneko, 1992). The logistic maps are coupled according to Equation 2, which describes the activity of map *i* at time *t* + 1 as a function of its activity at the previous time step and the activity of maps coupled with it. In this equation, *B*_*i*_ denotes the set of units connected to unit *i* (its neighbors in the network), and the coupling strength *ε*_*i*_ sets the proportion to which the average activities of coupled units influence the activity of unit *i* (Hellrigel et al., 2019).

$$x_{i,t+1}=\left(1-\varepsilon_{i}\right)\left(1-\alpha_{i}x_{i,t}^{2}\right)+\frac{\varepsilon_{i}}{\left|B_{i}\right|}\sum_{j\in B_{i}}\left(1-\alpha_{i}x_{j,t}^{2}\right).$$ (2)

Through the effect of the neighbors, the map activity of Figure S1C regains a noisy appearance more in line with Figure S1B. For efficiency of computation, we rewrite rewrite Equation 2 in matrix notation. For a network with *N* nodes, the activity of nodes at time *t* + 1 is calculated via Equation 3 (cf. the Supporting Information for its derivation).

$$X_{t+1}=\left[1_{N}-\alpha ⊙X_{t}⊙X_{t}\right]⊙\left[1_{N}-\varepsilon +\left(A_{t}\varepsilon \right)⊘\left(A_{t}1_{N}\right)\right].$$ (3)

In this equation, symbols ⊙ and ⊘ denote Hadamard (i.e., element-wise) multiplication and division, respectively. The right-hand side of Equation 3 constitutes the vector form of the logistic map, in which ***α*** is the vector of amplitudes and *X*_*t*_ is the vector of node’s activities at time *t*. The map is Hadamard-multiplied by a coupling term. In the coupling term, ***ε*** is the vector of coupling strengths, *A*_*t*_ is the connectivity matrix at time *t*, and **1**_*N*_ denotes a vertical unit vector of size *N*. In the coupling term, *A*_*t*_***ε*** is Hadamard-divided by *A*_*t*_**1**_*N*_, normalizing the former by the sum of the weights of the edges connected to each node. For binary networks, the term in the denominator counts the number of connections for each node. We consider only binary graphs for convenience. For adaptive rewiring in weighted networks, see Hellrigel et al. (2019).

Based on the network activity defined by Equation 3, adaptive rewiring takes the following form. After several updates to the network activity, a rewiring step is made. At each rewiring step, the connections of a random node are updated as follows: The node is disconnected from the neighbor most dissimilar in activity and is connected to the one most similar in activity among those it is presently unconnected to. Dissimilarity of two nodes at a given time is defined as the absolute value of the difference of their activity values. Note that although rewiring steps are local, the most dissimilar unconnected node is obtained through a global search. For algorithms using local or, instead, regional information for this purpose, see Jarman, Steur, Trengove, Tyukin, and van Leeuwen (2017); Jarman, Trengove, Steur, Tyukin, and van Leeuwen (2014).

All adaptive rewiring studies using coupled logistic maps have used fixed, uniform values for the amplitude parameters ***α*** and the coupling strengths ***ε***. In logistic map networks with fixed architectures, these parameters have previously been allowed to vary in order to implement information-processing functions. In a **perceptual organization** model (van Leeuwen, Steyvers, & Nooter, 1997), sensory input function was realized by local modulation of oscillator amplitudes. The presence of sensory input to certain units brought their amplitude parameter values down to impose a more stable regime on the oscillators. As a result, these oscillators showed a bias to become mutually synchronized. The synchronization was understood as representing perceptual grouping. Grouping preferences followed the Gestalt proximity principle and showed spontaneous switching in case of stimulation with bistable patterns.

In a memory model (van Leeuwen & Raffone, 2001), connectivity parameter values were locally incremented to represent the presence of a memory trace. This led to synchronization biases along the incremented connections, resulting in spontaneous rehearsal and relearning of stored pattern information. These examples demonstrate that coupled maps are capable of performing cognitive functions based on ongoing dynamics.

The question is whether the nonidentical parameter settings involved in these functions would dovetail with adaptive rewiring. To answer this question, we partitioned networks into two subsets of units, a majority and a minority, each receiving a different parameter value for the amplitude ***α*** or the coupling strength ***ε***. Fixing these parameters at different values involves a hardship test for adaptive rewiring. For instance, with one partition having increased coupling strength (or decreased amplitude), we might expect connections to be established preferentially within this partition and less within the other one. We may expect the opposite when coupling strength within the partition is decreased (or amplitude increased). In addition, we may expect fewer connections to be established between the partitions. This might induce network disintegration or otherwise interfere with the evolution of the network structure. On the other hand, if these perturbations fail to interfere with the emergence of complexity (i.e., small-wordness, modularity, and rich-club effect), adaptive rewiring could be used alongside pattern detection and learning in sparse neural networks. This has implications for the functioning of biological networks, as well as for the sparsification of artificial (i.e., deep) neural networks.

In what follows, the Method section describes details of the composition and initialization of the models, the rewiring algorithm, and the qualitative and quantitative measures of network structures used to describe, characterize, and compare models. In the Results section, we describe our findings, mainly that nonuniformity of parameters is shown not to interfere with the evolution of brain-like structure while giving rise to distinguishable network structures suitable for cognitive functions. A discussion and several concluding remarks end the paper.

## METHOD

### Description of Networks

An unlabeled binary graph (or network) *G* = (*V*, *E*) with *n* vertices (or nodes) and *m* edges (or couplings) is defined mathematically by a set of nodes *V* = {1, 2, …, *n*} and a set of edges *E* = {(*i*, *j*) ∈ *V*^2^ : *i* is coupled with *j*}. *E* is also known as the adjacency list of the network. We use undirected graphs, that is, (*i*, *j*) ∈ *E* ⇔ (*j*, *i*) ∈ *E*, and self couplings are not allowed, that is, (*i*, *i*) ∉ *E*. The size of the set *V* (i.e., the number of its members) is denoted by |*V*|. From *E*, we may construct the adjacency matrix *A* of *G* as a square matrix of the size |*V*|, the elements of which can take values of 0 or 1. The element on its *i*th row and *j*th column (i.e., *a*_*ij*_) is equal to 1 if (*i*, *j*) ∈ *E* and is 0 otherwise. Since *G* is undirected, *A* is symmetrical around the main diagonal, and since there are no self connections, its diagonal elements are zeros.

We may partition *V* into two nonoverlapping subsets, minority and majority, such that |*V*_*minority*_| = |*V*| − |*V*_*majority*_| and |*V*_*minority*_| ≪ |*V*_*majority*_|. The edges among members of these subsets form subgraphs within *G* and are henceforth called *minority* and *majority partitions*. A third subgraph comprises all of *V* but only edges between minority and majority nodes. Such a subgraph is called *interpartition*.

### Dynamics on the Graph

To each *v*_*i*_ ∈ *V*, an activation value is assigned according to Equation 3. The corresponding parameter values, that is, coupling strength ***ε*** and amplitude ***α***, remain fixed in our model simulations. Models with identical parameter sets are called *families*. Five families of models are simulated, each with 10 model instantiations, comprising a total of 50 model instantiations. Each network is run for 20 million iterations. All the simulations and analyses are conducted in R programming language version 3.6.0 (R Core Team, 2019) using computational resources provided by VSC (Flemish Supercomputer Center).

### Parameter Setting and Initialization

In our models, all *G*s have |*V*| = 300 nodes and 5,200 edges, a connectivity density providing robust evolution of small-world structure with uniform parameter setting (van den Berg, Gong, Breakspear, & van Leeuwen, 2012). The model structure is initialized by randomly assigning 5,200 × 2 = 10,400 values “1” symmetrically to nondiagonal entries of *A*, and zeros to the remaining entries. Each node in the network is randomly and independently assigned an initial value, uniformly distributed between 0 and 1, that is, *x*_*i*1_ ∼ *Unif* (0, 1).

Previous adaptive rewiring studies have been using values of ***α*** and ***ε*** in the ranges of [1.7–1.9] and [0.3–0.5], respectively (Gong & van Leeuwen, 2003; Hellrigel et al., 2019; van den Berg & van Leeuwen, 2004). Here the midpoints of these ranges, that is, ***α*** = 1.8 and ***ε*** = 0.4, are used for the parameters in the baseline (BL) condition. In the BL condition, all nodes have identical parameter values.

The same applies to the majority (250 nodes) of the other conditions. However, depending on the condition, the minority subset (i.e., the first 50) of nodes could have either lowered or increased values of either the ***α*** or ***ε*** parameters. As shown in Figure S2, higher values of the amplitude ***α*** tend to yield greater divergence in activity values; reducing the coupling parameter ***ε*** has a similar effect (Hellrigel et al., 2019). Conditions with lowered values of ***α*** are called less chaotic (LC), and those with increased values more chaotic (MC); conditions with lowered ***ε*** values are called sub-coupled (SC) and those with increased values hyper-coupled (HC). While keeping the parameters of the majority at the baseline level (*α*_*i*∈51:300_ = 1.8, *ε*_*i*∈51:300_ = 0.4), five different combinations of parameters were assigned to the minorities, each combination called a “family”: The baseline family (BL; *α*_*i*∈1:50_ = 1.8, *ε*_*i*∈1:50_ = 0.4), and the families with less chaotic minority (LC; ***α***_***i*∈1:50**_ = **1.7**, *ε*_*i*∈1:50_ = 0.4), more chaotic family (MC; ***α***_***i*∈1:50**_ = **1.9**, *ε*_*i*∈1:50_ = 0.4), sub-coupled minorities (SC; *α*_*i*∈1:50_ = 1.8, ***ε***_***i*∈1:50**_ = **0.3**), and hyper-coupled minority (HC; *α*_*i*∈1:50_ = 1.8, ***ε***_***i*∈1:50**_ = **0.5**). In the Results section, we identify model instantiations by the two capitals indicating their family, together with a serial number [1–10], for example, BL7, MC10. The 10 model instantiations within each condition are run with different initializations, which are identical across conditions to allow matched comparison between families.

### The Adaptive Rewiring Algorithm

A rewiring takes place after every 20 updates of the logistic maps, meaning that 1 million rewiring attempts are performed over the 20 million updates of the model. At each rewiring attempt, at time *t*, a node *i* is selected randomly from *V*, a vector of its distance from other nodes is calculated as ***d***_*i*,*t*_ = |*X*_*t*_ − *x*_*i*,*t*_**1**_|*V*|_|, and another vector of similarities is defined as ***s***_*i*,*t*_ = **1**_|*V*|_ − ***d***_*i*,*t*_.

Using these vectors, we compute two vectors: ***δ*** = [*δ*_1_, …, *δ*_*j*_, …, *δ*_|*V*|_]^*T*^ = *A***d**_*i*,*t*_ for the distances of node *i* from its neighbors; and ***σ*** = [*σ*_1_, …, *σ*_*j*_, …, *σ*_|*V*|_]^*T*^ = (*J*_|*V*|_ − *I*_|*V*|_ − *A*)***s***_*i*,*t*_ for the similarities of node *i* to its non-neighbors, where *J*_|*V*|_ is a |*V*| × |*V*| unit matrix (with all elements equal to 1) and *I*_|*V*|_ is the identity matrix of size |*V*| (with diagonal and off-diagonal elements equal to 1 and 0, respectively). The subtraction *J*_|*V*|_ − *I*_|*V*|_ − *A* ensures the search for the most similar node takes place among non-neighbors. The most dissimilar neighbor and the most similar non-neighbor of node *i*, respectively denoted as *ξ* and *ζ*, are marked by finding the index of the maxima of ***δ*** and ***σ***:

$$\begin{array}{l} \xi =\underset{j}{\text{argmax}}\;\delta_{j}\\ \zeta =\underset{j}{\text{argmax}}\;\sigma_{j}. \end{array}$$ (4)

The rewiring is then changing the corresponding elements of the adjacency matrix *A*:

$$\begin{array}{l} a_{i\xi }=a_{\xi i}=0\\ a_{i\zeta }=a_{\zeta i}=1. \end{array}$$ (5)

### Characterizing and Comparing Models

The state of each model at any given time *t* is described by adjacency matrix *A*_*t*_ (henceforth, “anatomical connectivity”), which is subject to adaptive rewiring, and the vector of activation values, *X*_*t*_. A model’s “functional connectivity” at *t* (represented by the |*V*| × |*V*| matrix *F*_*t*_) is defined by the momentary pairwise absolute differences of its node activation values.

#### Qualitative description of network structures.

Network structure can be qualitatively assessed by means of visual inspection of the wiring diagram or the adjacency matrix. Using the package *seriation* (Hahsler, Hornik, & Buchta, 2008), the adjacency matrix is serialized by ordering rows and columns according to the projection of the matrix on its first principal component. Seriation maximizes the visual identifiability of modules within the network.

#### Quantitative measures of the structure.

After each rewiring attempt, we calculate six network connectivity measures (Costa, Rodrigues, Travieso, & Villas Boas, 2007): edge density, clustering coefficient, average path length, small-world index, modularity, and assortativity. Furthermore, we calculate an additional measure, namely, the rich-club coefficient, after the final rewiring. All measures are calculated separately for the entire graph and the three subgraphs (viz., the minority, majority, and interpartition subgraphs).

##### Edge Density.

For a subset of nodes, this coefficient is the proportion of edges existing in a subgraph to the theoretical maximum number of edges possible in the same subgraph. For a subgraph with a subset of nodes |*V*_*s*_| and adjacency matrix *A*_*s*_, this value is calculated as

$$ED=\frac{\sum_{ij}A_{s}}{\left|V_{s}\right|.\left(\left|V_{s}\right|-1\right)}.$$ (6)

Since the total number of edges remains the same during the adaptive rewiring, this coefficient gives an indication of how strongly each partition has attracted new nodes at every rewiring step.

##### Clustering Coefficient.

This measure can be defined either locally or globally and gives an indication for the tendency of nodes to form clusters. We use the global clustering coefficient, which is defined as the number of closed triplets of nodes (the triplets of nodes that are all connected) divided by the number of connected triplets, either open (i.e., paths of length two) or closed (i.e., triangles). The numerator is equal to 3 times the number of triangles in the graph. Using linear algebra, the global clustering coefficient can be calculated formally from the adjacency matrix *A* via

$$C=\frac{3\times \text{\#}\;\text{triangles}}{\text{\#}\;\text{triplets}\;\text{of}\;\text{connected}\;\text{nodes}}=\frac{Tr\left(A^{3}\right)}{\sum_{ij}}A^{2}-Tr\left(A^{2}\right).$$ (7)

In this equation, *Tr*(*A*) is the trace of matrix *A* and is defined as the sum of its diagonal elements, that is, *Tr*(*A*) = ∑_*i*_ *A*_*ii*_.

##### Average Path Length.

The average path length is the mean value of lengths of shortest paths between all pairs of nodes, as defined in Equation 4 for a network of size *N*, where *d*_*ij*_ is the length of the shortest distance between nodes *i* and *j*, and *d*_*ij*_ = 0 if there is no path between *i* and *j*. This measure, which we calculated using the *igraph* package (Csardi & Nepusz, 2006), gives an indication of how closely the nodes of a network are located from each other.

$$PL=\frac{1}{N\left(N-1\right)}\sum_{i\neq j\in V}d_{ij}.$$ (8)

##### Small-world Index.

This measure quantifies the degree to which a graph shows the optimal combination of local and global connectedness, known as *small-worldness* (Watts & Strogatz, 1998). It is defined as the proportion of clustering coefficient to average path length for a network,

$$SW=\frac{C}{PL}.$$ (9)

The small-world index is often normalized by *SW*_0_ = $\frac{C_{0}}{{PL}_{0}}$, the expected small-world index of a random network of the same size and density as the network in question. *C*_0_ and *PL*_0_ are, respectively, the expected clustering coefficient and expected average path length in such a random network. Thus, the normalized small-world index is given by

$${SW}_{\mathrm{norm}}=\frac{SW}{{SW}_{0}}=\frac{\frac{C}{PL}}{\frac{C_{0}}{{PL}_{0}}}.$$ (10)

The normalized index makes the comparison of networks with different sizes and densities possible. Since all networks modeled in this study start off with random networks of equal size and density, for computational reasons, the non-normalized small-world index (Equation 9) is calculated and reported.

##### Modularity.

Modularity of a network, as proposed by Newman (2006) and denoted by *Q*, is a measure of to what extent the nodes tend to form interconnected communities isolated from the other nodes of the graph. More precisely, for a network of size *N* (with the theoretical maximum number of edges *m* = $\frac{N\left(N-1\right)}{2}$) and adjacency matrix *A*, modularity is defined as

$$Q=\frac{1}{2m}\sum_{i,j}\left[M_{ij}-\frac{k_{i}k_{j}}{2m}\right]\delta \left(c_{i},c_{j}\right).$$ (11)

In this equation, *k*_*i*_ and *k*_*j*_ are, respectively, degrees of nodes *i* and *j*. *δ*(*c*_*i*_, *c*_*j*_) is the Kronecker delta function, which is equal to 1 if nodes *i* and *j* have the same label and 0 otherwise. The term in square brackets is the difference between the actual number of edges between nodes *i* and *j* and the expected number of edges between them. Hence, according to Equation 11, modularity equals the sum of these differences for the nodes within communities, normalized by the theoretical maximum number of edges in the network.

This measure requires an a priori labeling of nodes that defines the communities to which the nodes belong. A variety of algorithms have been suggested to discover module, or communities, within a network so that the value of *Q* is maximized (for a review, cf. Zhang, Ma, Zhang, Sun, & Yan, 2018). The communities discovered by these algorithms can thus be used as labels for calculating modularity of the network. In line with Clauset, Newman, and Moore (2004), we use the fast greedy algorithm to optimally detect communities and thus calculate the modularity based on community membership of the nodes. The *igraph* package was used to automatically detect communities and calculate modularity.

##### Assortativity.

The assortativity coefficient is a measure of homophily in networks that indicates the preferences of nodes to connect to alike nodes. The likeness can be imposed externally, for example, by assigning categories to the nodes using labels (known as nominal assortativity), or by internal criteria such as node degrees (degree assortativity). Degree assortativity is defined as the Pearson correlation coefficient of degrees of connected nodes, thus taking values in the range [−1, 1].

To give a formal definition, let *p*_*k*_ be the probability that a randomly chosen node has degree *k*. It can be shown that the degree distribution for a node connected to a randomly selected edge *l* is thus proportional to *kp*_*k*_. Then, a quantity for “remaining degree” is defined as *q*_*k*_ = $\frac{\left(k+1\right)p_{k+1}}{\sum_{j}{\mathrm{Tj}p}_{j}}$, which is the normalized distribution of remaining degree for the nodes connected to *l*. Finally, the joint probability of remaining degrees of the nodes at both ends of *l* is denoted by *e*_*jk*_. For an undirected network *e*_*jk*_ = *e*_*kj*_ and its marginal distribution is ∑_*j*_ *e*_*jk*_ = *q*_*k*_. Having the variance of remaining degree as $\sigma_{q}^{2}$ = ∑_*k*_ *k*^2^*q*_*k*_ − [∑_*k*_ *kq*_*k*_]^2^, the degree assortativity can be calculated as shown in Equation 12 (Newman, 2003).

$$r=\frac{1}{\sigma_{q}^{2}}\sum_{ij}jk\left(e_{jk}-q_{j}q_{k}\right).$$ (12)

##### Rich-club Coefficient.

This coefficient quantifies the tendency of nodes with higher than a certain degree to connect to each other. More formally, as Equation 13 shows, the coefficient is equivalent to the edge density of the subgraph of the network where the nodes with lower degrees than the cutoff value *k* are removed,

$$RC\left(k\right)={ED}_{\geq k}=\frac{\sum_{ij}A_{\geq k}}{N_{\geq k}\left(N_{\geq k}-1\right)}.$$ (13)

Since this coefficient is a function of club size *k*, it is hard to visualize its evolution over time for all possible values of *k*. Hence, the values of this coefficient were only plotted for the final state of the networks.

The absolute value of the rich-club coefficient is hard to interpret and is not comparable among networks of different sizes, densities, and degree distributions. Hence, this coefficient is often normalized by the average rich-club coefficient of random networks of the same size with similar **degree sequence**. For each model (and its minority and majority partitions), we simulated 200 such networks, for each club size *k*, and normalized the values of the rich-club coefficients of the network by the average rich-club coefficient among the random networks. Moreover, for each *k*, we statistically tested whether the non-normalized *RC* is significantly larger than the rich-club coefficients of randomly generated networks. Since the distribution of *RC*s of random networks were non-Gaussian, the conventional one-sample *t* test was not applicable. Instead, we performed one-sample Wilcoxon signed rank test and set *α* = 0.01 as the significance level for the *p* values.

For a certain *k*, a normalized rich-club coefficient larger than 1 indicates that nodes with degree *k* tend to connect to the “rich” nodes (i.e., those with degrees equal to or greater than *k*), thus forming “rich clubs.” Conversely, *RC*_*norm*_(*k*) < 1 implies that the nodes with degree *k* show a tendency to connect to nodes with lower degrees. Finally, *RC*_*norm*_(*k*) = 1 suggests that nodes with degree *k* show no preference to connect to nodes with lower or higher degrees.

#### Investigating the resemblance between models.

To compare network families with each other, we assume, in line with Berlingerio, Koutra, Eliassi-Rad, and Faloutsos (2012), that the structural information embedded in networks can be summarized by the distributions of local network measures. Comparison of networks is thus reduced to comparing these distributions. To obtain measures of distributional distances, we use the NetSimile method (Berlingerio et al., 2012) and the Heller–Heller–Gorfine (HHG) algorithm (Heller, Heller, & Gorfine, 2013), both of which are discussed in the Supporting Information. In short, NetSimile indicates the degree of dissimilarity between the distributions attributed to the networks being compared via comparing signature vectors that encapsulate the distributions by their summary statistics.

HHG, on the other hand, provides *p* values for a test of independence among the distributions; a small *p* value derived from HHG (e.g., below the threshold of *α* = 0.05) provides evidence to reject the null hypothesis of distributional independence. Thus, loosely speaking, HHG *p* value can be regarded as an indicator for dissimilarity; a higher value of this measure entails a smaller “resemblance” (or dependence) between the networks. Nevertheless, interpreting HHG *p* values as such a measure is somewhat unorthodox and is hardly meaningful unless used along with another dissimilarity measure such as NetSimile.

We first make pairwise comparisons among the 1,225 unique pairs of model instantiations at their final state after 1 million rewiring attempts. Having quantitative measures for dissimilarities among the networks, we quantify the within-family resemblances and between-family contrasts among the models. Finally, we define a score for family distinction in order to compare how families vary with respect to this measure.

##### Family Resemblances and Differentiations.

The outcomes of pairwise comparison of the networks using NetSimile and HHG were stored in four 50 × 50 matrices of form ${\text{Dissimilarity}}_{M}^{G}$ for networks *G* (either *A* or *F*, respectively for anatomical and functional connectivities) based on method *M* (either NetSimile or HHG). To ease the visual comparison of these measures, the matrices of NetSimile distances, that is, ${\text{Dissimilarity}}_{\text{NetSimile}}^{A}$ and ${\text{Dissimilarity}}_{\text{NetSimile}}^{F}$, were normalized by the highest value in each matrix so their values range from 0 to 1. Then, within- and between-family contrast aggregate scores were calculated by averaging the elements of dissimilarity matrices that belong to the families being compared as shown in Equation 14:

$${\text{Contrast}}_{M}^{G}\left(f_{i},f_{j}\right)=\frac{1}{10\times 10}\sum_{i\in f_{i}}\sum_{j\in f_{j}}{\left({\text{Dissimilarity}}_{M}^{G}\right)}_{ij}.$$ (14)

Finally, a differentiation score was calculated for each family to quantify the degree to which models belonging to family *f*_*i*_ resemble each other and, at the same time, diverge from the members of other families, via

$${\text{Differentiation}}^{G}\left(f_{i}\right)=\frac{1-{\text{Contrast}}_{\text{NetSimile}}^{G}\left(f_{i},f_{i}\right)}{\frac{1}{4}\sum_{i\neq j}\left[1-{\text{Contrast}}_{\text{NetSimile}}^{G}\left(f_{i},f_{j}\right)\right]}.$$ (15)

In this equation, the numerator is the within-family resemblance of networks belonging to family *f*_*i*_. The denominator is the mean of the between-family resemblance of *f*_*i*_ to other families. This score will be larger than 1 if family *f*_*i*_ differentiates from other families. For the lack of straightforward interpretation, this score was not calculated for HHG outcomes.

## RESULTS

### Network Structures

**Figure 1.**
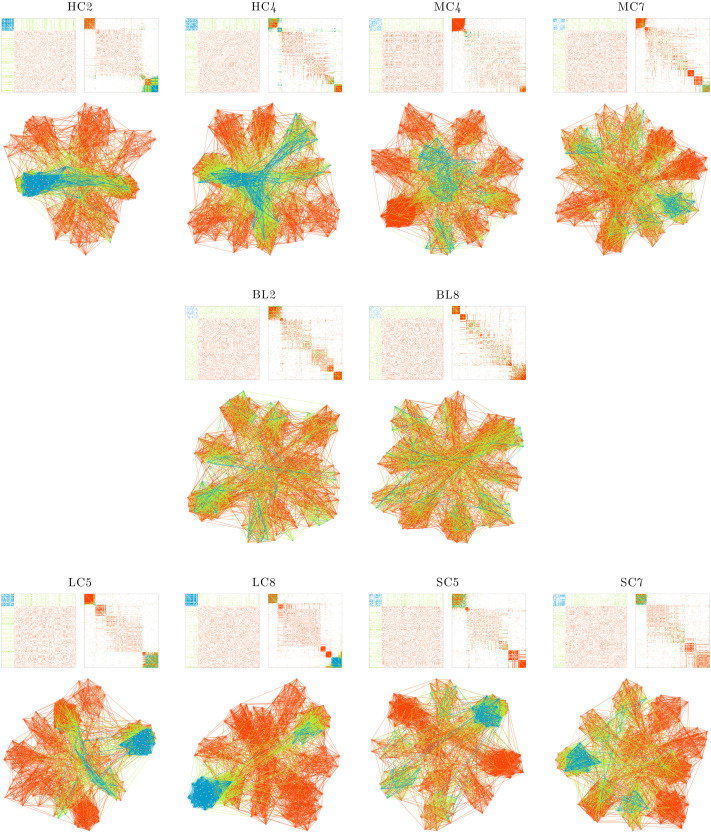
Network structures of representative models. Each panel shows the unserialized (top left) and serialized (top right) adjacency matrices, and the graph representation (bottom) of the structural connectivity at the last rewiring step. The within-minority, within-majority, and interpartition edges are colored blue, red, and green, respectively. In the graph representation, the minority and majority nodes are colored sky blue and pink, respectively.

Using the *seriation* and *igraph* packages, we plot the raw (unserialized) and ordered (serialized) adjacency matrices and the wiring diagrams of the networks in their final state. In the plots, the minority and majority nodes are colored sky blue and pink, respectively. In both matrix visualizations and wiring diagrams, the within-minority and within-majority edges are colored blue and red, respectively. The interpartition edges, connecting nodes of minority subset to nodes of majority, are colored green. Although there are variations among models, either within- or between-families, in all models (except for the MC2, MC3, SC1, and SC3; see below), several densely coupled sets of nodes, that is, modules, emerged. These modules can be identified as squares in the serialized adjacency matrices. Figure 1 shows two representative networks per family. As evident in the plots, the networks manifest a range of different structures. Yet, similarities can be observed among models belonging to the same families.

The baseline models (BL2 and BL8 in Figure 1) typically include three densely coupled modules and a few larger, sparser sets of nodes. The modules are not isolated from the rest of the network, as intermodular edges keep them connected to other nodes. The unserialized adjacency matrices show that the density of edges is relatively uniform over subsets of nodes.

In the HC family, wherein the minority nodes have relatively higher coupling strengths, the edge density is higher in the minority subgraph. Moreover, the HC family networks have more distinct modules than other families. HC2 (Figure 1), for instance, has only two modules, both of which are highly connected. Higher edge density in the minority subset and highly distinct modules are also observed for the LC family, which has lower amplitude in the minority nodes (LC5 and LC8, Figure 1). The similarity between the HC and LC models was expected, as the nodes with lower amplitudes and higher coupling strengths can synchronize more easily. However, it is worth noting that the effect is not limited to the minority nodes; highly connected modules also emerge among the majority nodes.

In the MC family, the edge density in the minority partition is reduced. Moreover, the higher level of amplitude for minority nodes resulted in highly connected modules among the majority nodes (see MC4 in Figure 1). In the SC family, the edge density of the minority (which had lower coupling strengths) is lower than that of the majority. Additionally, the lower coupling strength of the minority prevented minority nodes from forming modules, and they were absorbed into modules formed mainly by the majority nodes. See SC5 and SC7 in Figure 1.

### Network Statistics

**Figure 2.**
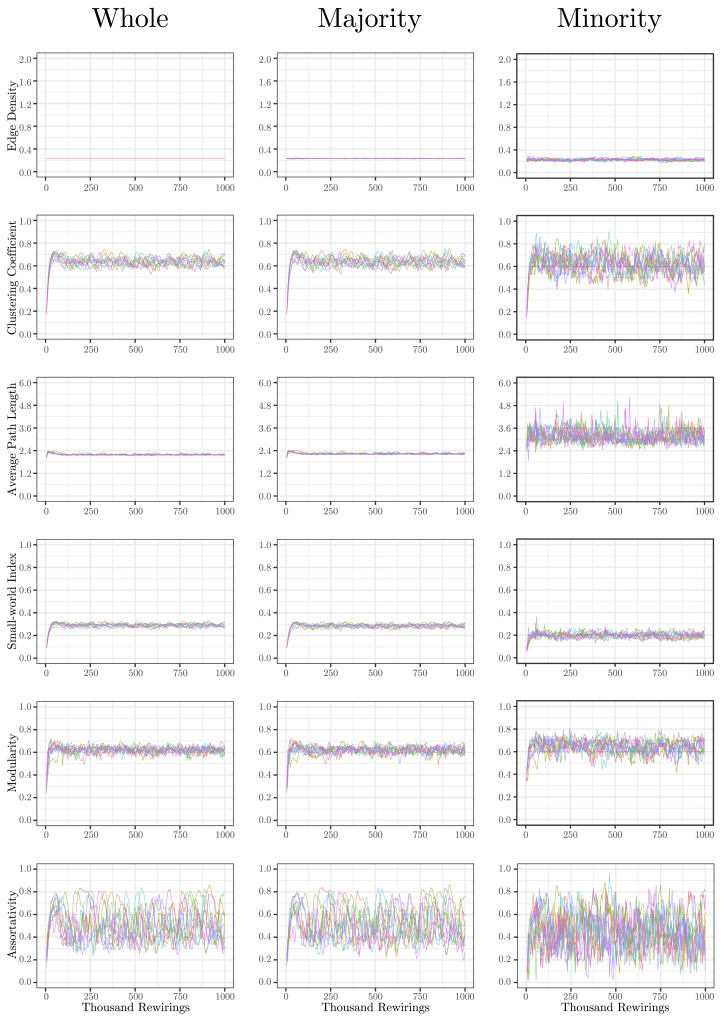
Evolution of network statistics in the baseline (BL) condition for the whole network and majority and minority subgraphs.

**Figure 3.**
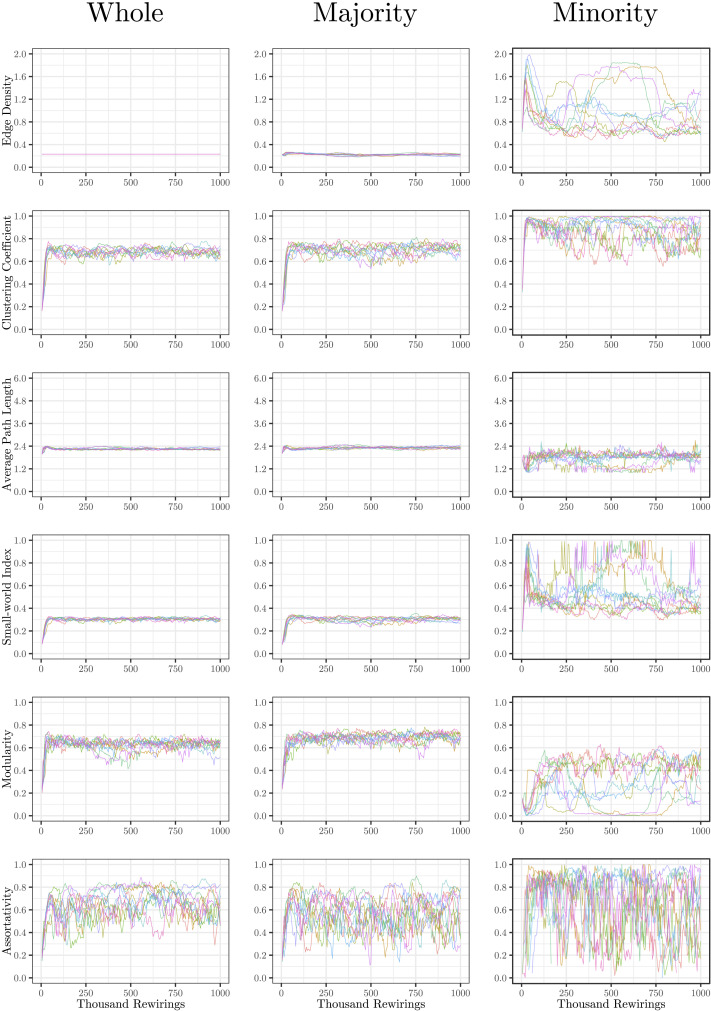
Evolution of network statistics in the less chaotic (LC) condition for the whole network and majority and minority subgraphs.

**Figure 4.**
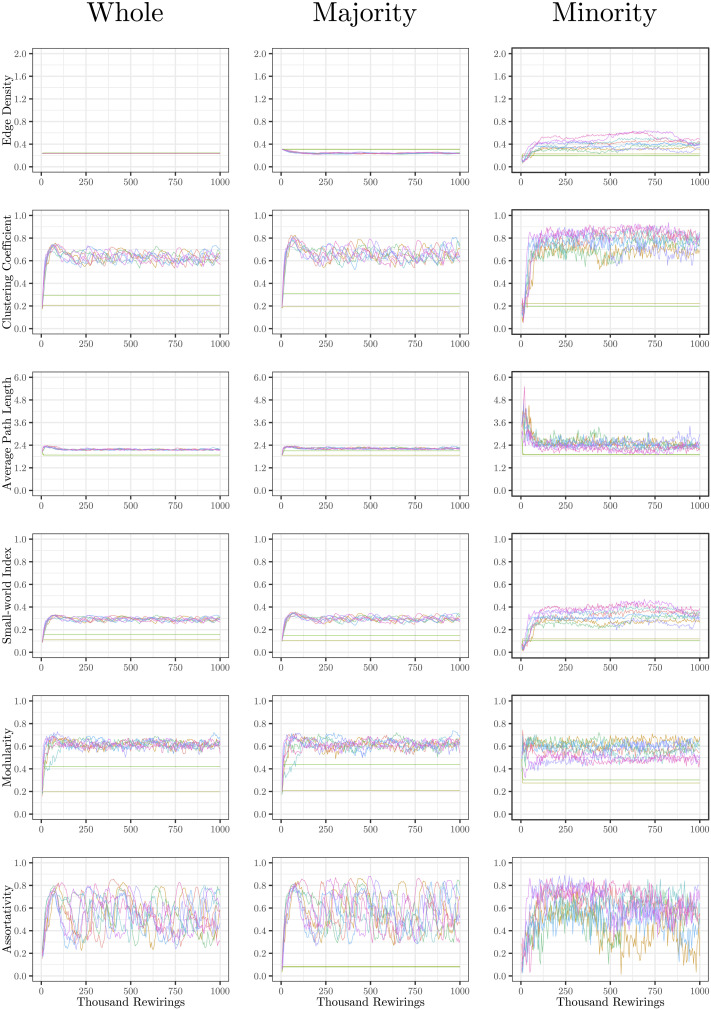
Evolution of network statistics in the more chaotic (MC) condition for the whole network and majority and minority subgraphs.

**Figure 5.**
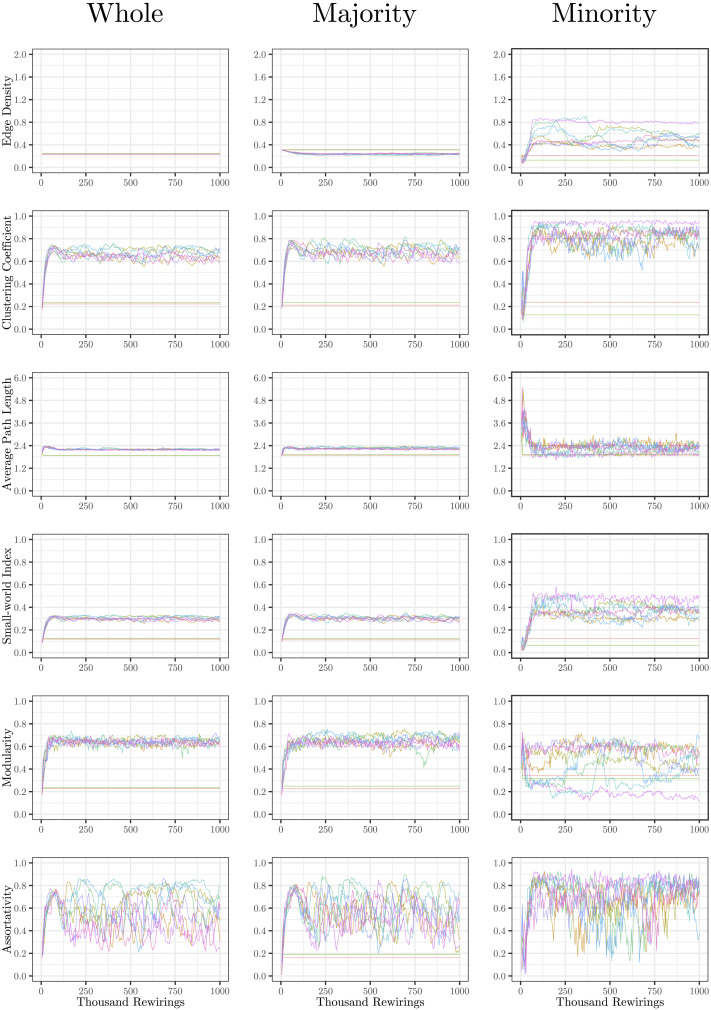
Evolution of network statistics in the sub-coupled (SC) condition for the whole network and majority and minority subgraphs.

**Figure 6.**
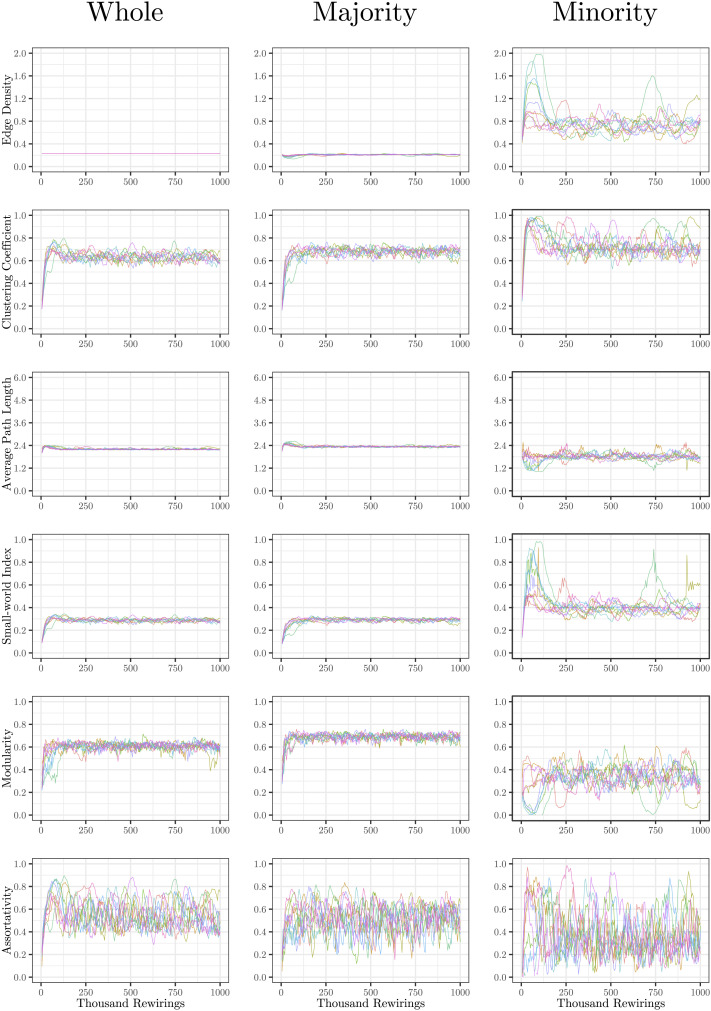
Evolution of network statistics in the hyper-coupled (HC) condition for the whole network and majority and minority subgraphs.

**Figure 7.**
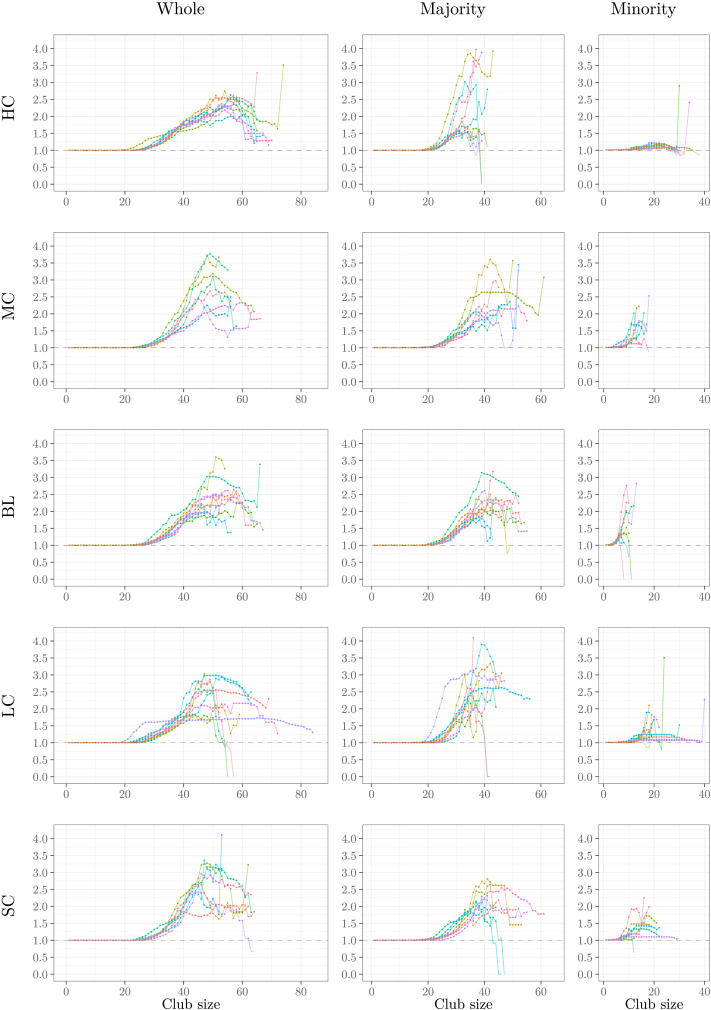
Normalized rich-club coefficients of the whole network after the last rewiring step, grouped by condition. Solid circles mark significant values.

The evolution of clustering coefficient, modularity, edge density, small-world index, degree assortativity, and average path length were plotted for the structural network connectivity of all models and their subnetworks (viz., within-minority, within-majority, and interpartition). Figures 2–6 show these plots for the minority, majority, and whole networks, grouped per family. Furthermore, the normalized rich-club coefficient of the final states of the minority, majority, and whole networks are plotted in Figure 7.

Let us first consider the evolution of network statistics for the whole network. As evident in the plots, modularity, clustering coefficients, and small-world index of all models increase noticeably after 60,000 rewiring attempts (roughly by a factor of 4.7, 5.3, and 4.6, respectively) and degree assortativity approaches 0.53. Meanwhile, the average path length increases no more than 15%. The network statistics fluctuate afterward, and the fluctuation is more pronounced for degree assortativity. The majority subnetworks show similar trends. In the minority subnetworks—although with higher fluctuations and instability in HC, LC, and SC—we observe an increase in these statistics after 60,000 rewiring attempts. The network statistics of minority subnetworks of MC and BL families resemble those of the whole networks in the same families. Yet, clustering coefficient and small-world index show higher growth in the minority subnetworks (more than six- and sevenfold, respectively). The exception to these improvements is the modularity, which increases only moderately (roughly 35%) in this subnetwork. Given the finite number of nodes in this partition, this was not unexpected; a larger subnetwork would likely have shown more improvement in modularity as well. The edge densities in the minority subnetworks, although unstable, are often large, roughly 2.4 times that of the whole network. In line with elevated edge density, average path length within the minority subgraph drops by almost 7%. This means that the minority nodes tend to connect more strongly to each other than to other nodes. Tables 1–3 summarize the mean and standard deviation of changes in network statistics after 60,000 rewiring attempts for the whole network and the majority and minority subnetworks.

**Table 1.** Means and standard deviations (between parentheses) of network statistics of the whole graph for different conditions. Means are from 60,000 to 1 million rewiring steps, normalized by those of 100 random networks, except degree assortativity. BL: baseline; LC: less chaotic; MC: more chaotic; SC: sub-coupled; HC: hyper-coupled.

Condition	Clustering coefficient	Average path length	Small-world index	Modularity	Assortativity	Edge density
HC	5.33 (1.05)	1.15 (0.05)	4.62 (0.80)	4.69 (0.85)	0.52 (0.22)	1 (0.02)
MC	5.34 (1.02)	1.15 (0.05)	4.63 (0.78)	4.7 (0.81)	0.53 (0.22)	1 (0.01)
BL	5.32 (1.05)	1.14 (0.05)	4.62 (0.80)	4.68 (0.84)	0.53 (0.22)	1 (0.02)
LC	5.35 (1.03)	1.15 (0.05)	4.63 (0.79)	4.7 (0.82)	0.53 (0.22)	1 (0.02)
SC	5.35 (1.01)	1.15 (0.05)	4.64 (0.78)	4.69 (0.82)	0.53 (0.22)	1 (0.01)

**Table 2.** Means and standard deviations (between parentheses) of network statistics of the majority subgraph for different conditions. Means are from 60,000 to 1 million rewiring steps, normalized by those of 100 random networks, except degree assortativity. BL: baseline; LC: less chaotic; MC: more chaotic; SC: sub-coupled; HC: hyper-coupled.

Condition	Clustering coefficient	Average path length	Small-world index	Modularity	Assortativity	Edge density
HC	5.49 (1.13)	1.17 (0.06)	4.64 (0.85)	4.45 (0.84)	0.52 (0.18)	1.01 (0.12)
MC	5.51 (1.08)	1.17 (0.06)	4.66 (0.82)	4.47 (0.8)	0.52 (0.18)	1 (0.11)
BL	5.49 (1.12)	1.17 (0.06)	4.64 (0.85)	4.44 (0.83)	0.52 (0.18)	1.01 (0.12)
LC	5.52 (1.11)	1.18 (0.06)	4.66 (0.84)	4.47 (0.81)	0.53 (0.18)	1 (0.12)
SC	5.53 (1.08)	1.18 (0.06)	4.67 (0.83)	4.46 (0.8)	0.54 (0.18)	1 (0.12)

**Table 3.** Means and standard deviations (between parentheses) of network statistics of the minority subgraph for different conditions. Means are from 60,000 to 1 million rewiring steps, normalized by those of 100 random networks, except degree assortativity. BL: baseline; LC: less chaotic; MC: more chaotic; SC: sub-coupled; HC: hyper-coupled.

Condition	Clustering coefficient	Average path length	Small-world index	Modularity	Assortativity	Edge density
HC	6.24 (1.71)	0.94 (0.24)	7.16 (3.19)	1.35 (0.56)	0.53 (0.32)	2.36 (1.44)
MC	6.26 (1.69)	0.93 (0.24)	7.27 (3.24)	1.35 (0.57)	0.53 (0.31)	2.42 (1.49)
BL	6.18 (1.72)	0.94 (0.24)	7.07 (3.03)	1.38 (0.54)	0.52 (0.32)	2.35 (1.39)
LC	6.19 (1.70)	0.93 (0.23)	7.11 (3.11)	1.39 (0.52)	0.52 (0.32)	2.34 (1.38)
SC	6.2 (1.67)	0.93 (0.24)	7.17 (3.25)	1.37 (0.55)	0.51 (0.31)	2.36 (1.47)

Figure 7 shows the normalized rich-club coefficient *RC*_*norm*_(*k*) of the minority, majority, and whole networks, grouped by families, as a function of club size *k*. *RC*_*norm*_(*k*) above 1 (dashed line) indicates rich clubs. Values significantly larger than 1 (*p* < 0.01, based on one-sample Wilcoxon signed rank test) are marked by solid circles. Despite differences among families, they all show significant rich-club structures for larger club sizes, both in the whole network and in the majority partition. Let us first consider the plots for the whole networks, depicted in the left column of this figure. The HC family has clubs of sizes 30 < *k* < 70 (involving 10–23% of the nodes), most of them significant, with consistent *RC*_*norm*_ values among members. In the LC family, all models form rich clubs with 25 < *k* < 55 (roughly 8–18% of the nodes), and some models form larger rich clubs up to the sizes of 65–85 (roughly 22–28% of the nodes). Other families, that is, MC, BL, and SC, form clubs with sizes ranging from 30 to roughly 65 (10–22% of the nodes), with relatively higher (but less consistent) *RC*_*norm*_ values compared with HC.

In the majority partition (middle column of Figure 7), we consistently observe rich clubs larger than 25, involving 10% of the nodes within that partition. The HC family has rich clubs as large as 40 (16% of nodes), and other families form even larger rich clubs, with sizes spanning 45 to 60 (making up 18–24% of the nodes). In general, we observe more diverse values among models for the rich-club coefficients in the majority partition. Finally, in the minority partition (the right column of the same figure), we observe a relatively remarkable—and consistent—rich-club effect in the HC family (12 < *k* < 30; 24–60% of nodes), while other families have relatively larger rich-club coefficients for smaller club sizes (5 < *k* < 20; 10–40% of nodes). Some models of the LC family form much larger rich clubs with sizes up to 40 (i.e., 80% of the nodes).

### Incidental Losses

**Figure 8.**
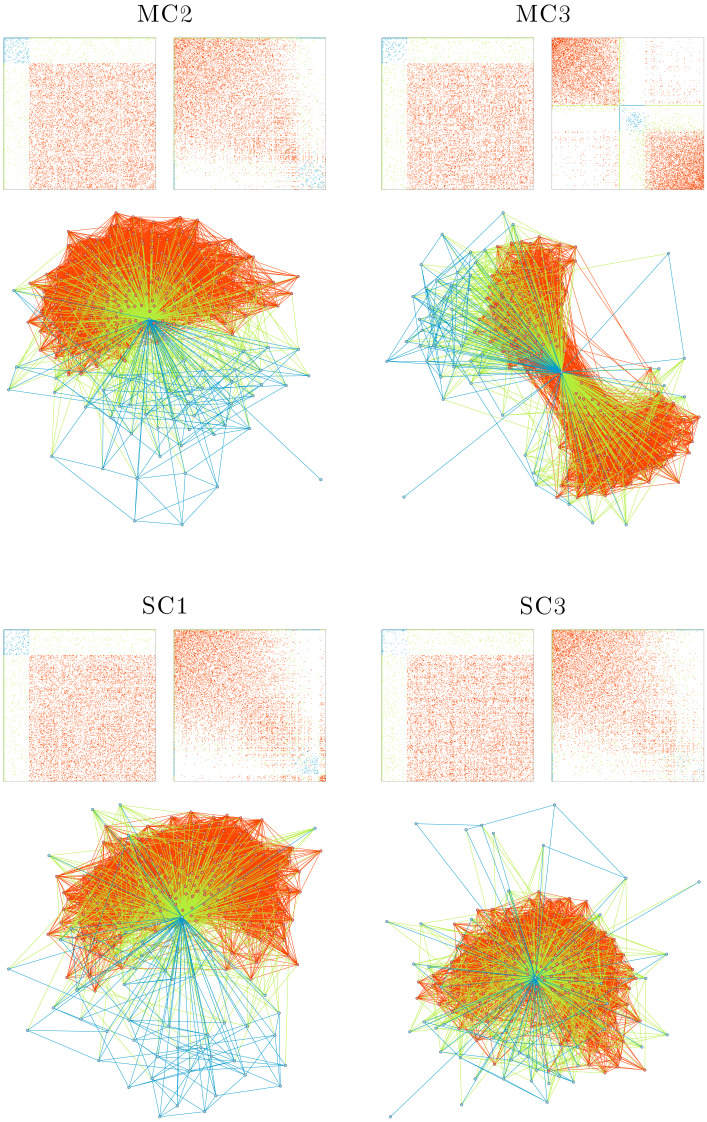
Network structures of terminated models. Panels and color coding are similar to those of Figure 1.

As can be observed in the evolution plots, four models (viz., MC2, MC3, SC1, and SC3) stop evolving before 10,000 rewiring attempts. Visual inspection reveals that, at some point in their evolution, one node reaches maximum degree and is connected to all other nodes (cf. Figure 8). This indicates that these models reached the end point of a pathological development, something we chose not to prevent in our algorithm. Consequently, the rewiring algorithm naturally terminates, as division by 0 looms in Equation 3. Terminated models failed to form any modules and were omitted from family-wise comparisons. We consider these incidental losses as a sign that robustness against perturbation is not always guaranteed for our models.

### Family-Wise Comparisons

NetSimile and HHG similarity measures were composed in new matrices wherein the lower triangle belongs to ${\text{Dissimilarity}}_{HHG}^{N}$ and the upper triangle belongs to ${\text{Dissimilarity}}_{\text{NetSimile}}^{N}$, that is,

$${\text{Dissimilarities}}^{N}=\text{lower}.\text{tri}\left({\text{Dissimilarity}}_{HHG}^{N}\right)+\text{upper}.\text{tri}\left({\text{Dissimilarity}}_{\text{NetSimile}}^{N}\right).$$ (16)

**Figure 9.**
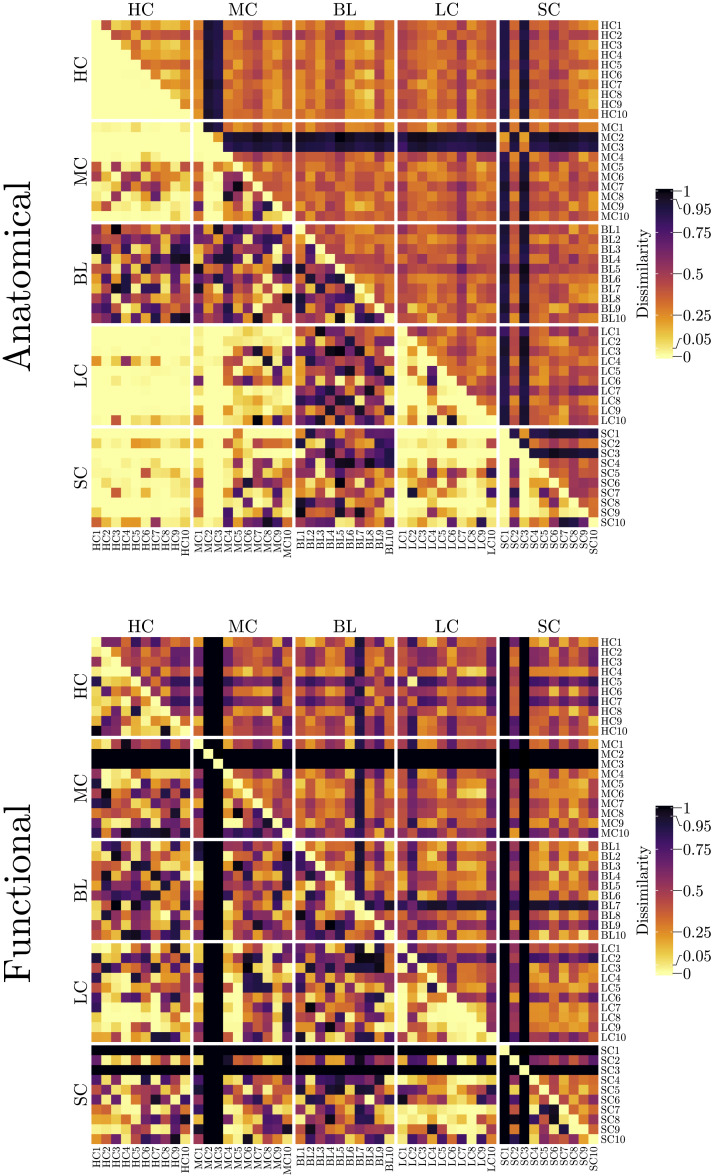
Heat maps of pairwise dissimilarities of anatomical (top) and functional (bottom) networks. The upper diagonal elements show normalized dissimilarity measures derived from NetSirnile algorithm, and the lower diagonal elements show HHG *p* values. Model names and family assignments are indicated. Lower dissimilarity (hence higher similarity) measures are coded by brighter colors.

The matrices of *Dissimilarities*^*N*^ are plotted in Figure 9 as heat maps using the *ComplexHeatmap* R package (Gu, Eils, & Schlesner, 2016).

**Figure 10.**
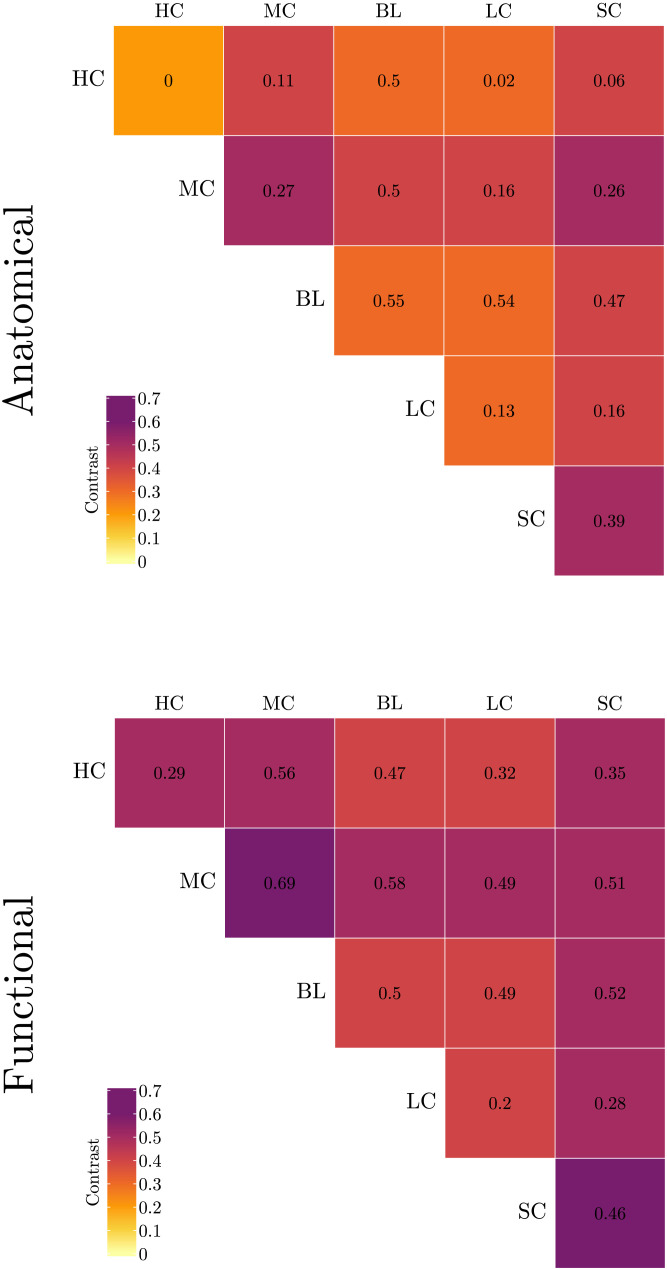
Heat maps of within- and between-family contrasts for anatomical (top) and functional (bottom) connectivities. The values within cells show the average HHG *p* values of corresponding family-wise comparisons. Lower contrast measures are coded by brighter colors.

The matrices of *Contrast*^*N*^ are plotted as upper triangular matrices in Figure 10 using the *corrplot* R package (Wei & Simko, 2021). The cell colors, coded similarly to the heat maps, denote average contrast measures derived from the NetSimile algorithm while average HHG *p* values (i.e., ${\text{Contrast}}_{HHG}^{N}$) are indicated in each cell. The HC family manifests the least within-family contrast. Based on the HHG test of multivariate independence, except for HC-HC and HC-LC family pairs, no conclusive evidence exists for distributional dependence among families. Finally, as can be seen in this figure, the within- and between-family NetSimile contrasts of both structural and functional connectivity networks show similar patterns. More specifically, both the structural and the functional connectivity of HC-BL, HC-LC, BL-BL, BL-LC, and LC-LC all share close contrast values compared with other family pairs. This is also the case for the BL-SC and LC-SC pairs.

**Figure 11.**
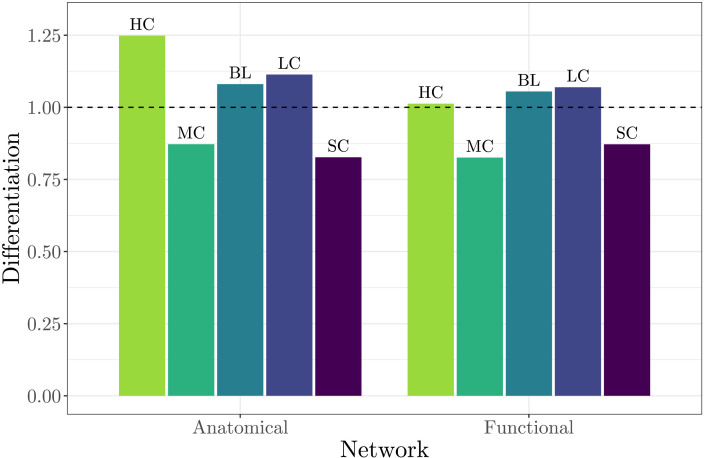
Between-family differentiation scores of the anatomical and functional networks. Values above 1 (dashed line) imply above-average within-family resemblance compared with other families.

The differentiation scores for structural and functional connectivity between families are plotted in Figure 11. Differentiation values above 1 (dashed line) imply that the within-family resemblance of network structures of family *f*_*i*_ is higher than the average resemblance of its members to the members of other families. We observe elevated differentiation in both structural and functional networks of HC, BL, and LC. This measure is remarkably higher for the structural networks of the HC family while barely exceeding the threshold for the functional network of the same family.

**Figure 12.**
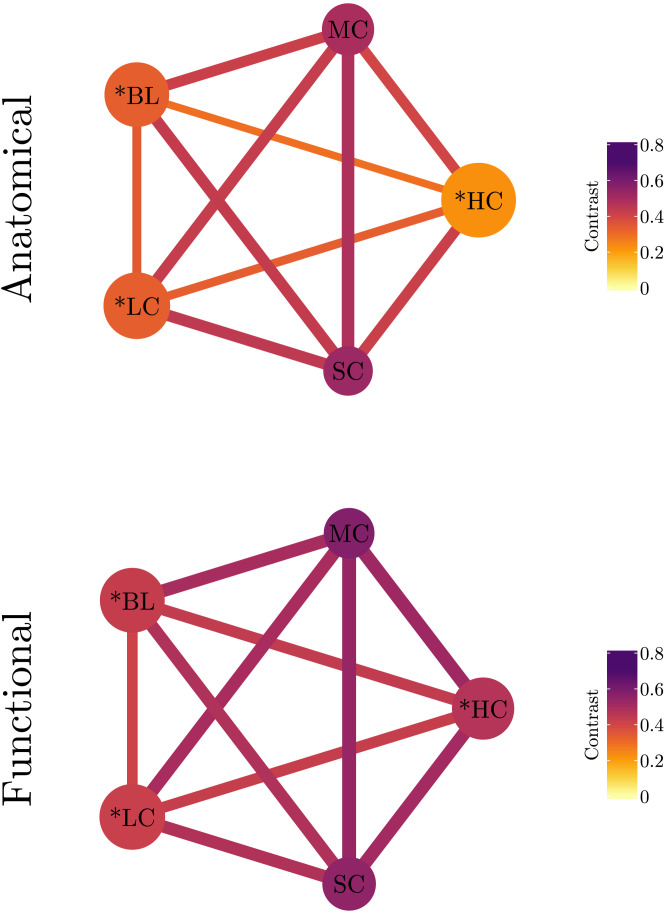
Graph representation of family resemblance and differentiation in anatomical (top) and functional (bottom) connectivities of the fully evolved models. Edge color and size code between-family contrast and node color captures within-family contrast. Node size is proportional to the differentiation score of the corresponding family. The families with ditferentiation scores above 1 are marked with asterisks.

Finally, in order to have both family resemblance and family differentiation in a single frame, we summarized their values in the graphs shown in Figure 12. In these graphs, individual nodes represent families of models. Edge color and size code between-family contrast and node color captures within-family contrast. The size of each node is proportional to the value of the differentiation score of its corresponding family. The families with *Differentiation*^*N*^(*f*_*i*_) > 1 are marked with asterisks. It can be noticed that in both structural and functional networks, the families with differentiation scores larger than 1 (i.e., HC, BL, and LC) have lower within-family contrast values. Moreover, the pairwise contrasts among these families (i.e., HC-BL, HC-LC, and BL-LC) are lower than any other family pair. This suggests that models with increased amplitude and decreased coupling strengths lead to structures less resembling the baseline family.

## DISCUSSION

We investigated the effect of nonuniformity of oscillator amplitude and connection strength on the evolution of network structure under adaptive rewiring. The adaptive rewiring was driven by synchronization in coupled logistic maps. The amplitude and coupling parameters of the oscillators govern their synchronization behavior. It has amply been shown that networks evolve to complex, brain-like structures when these parameters were fixed to uniform values. We replicated this behavior for our baseline parameter setting. Additionally, we studied how nonuniform parametrization gets reflected in the evolving network structure and whether adaptive rewiring is robust to these perturbations.

To this aim, a minority subset of network units had either the amplitude reduced (LC) or increased (MC), or the connectivity reduced (SC) or increased (HC). These perturbations may be compared with perceptual input (modeled by increased or decreased divergence in node activities) or memory processes (modeled by varying degrees of coupling). In all these conditions, adaptive rewiring establishes the evolution of random networks into complex structures. These structures are modular and small-world networks and show a fivefold increase in the clustering coefficient, at the cost of an increase of less than 15% in average path length compared with the original random networks. Moreover, the models form rich clubs as large as 8–28% of network size. This evolution is in accord with previous adaptive rewiring studies. Adaptive rewiring, therefore, is generally robust against the symmetry-breaking perturbations of system parameters and thus can accommodate—and integrate—function-specific modules. Note, however, that robustness is not always guaranteed. In a small proportion of our models (four cases in total), we observed pathological development in the perturbed models, resulting in network evolution breakdown.

On top of these findings about the whole network, the subsets of our models also developed brain-like structures. More specifically, across families, the majority subnetwork shows improved properties akin to the whole network. The minority subnetwork of perturbed models—with an exception for modularity—manifests these properties to a much greater degree, especially in the relative sizes of its rich clubs. The latter observation is indicative of more specialized structures in the minority subnetwork as a direct result of heterogeneity in model parameters.

Despite the overall consistency in structural improvements of networks, we observed considerable variability among models, both in their evolution and in the final states of structural and functional connectivity. These variations are partly due to the family-specific parameterization of models and partly due to the random initialization of the models. We quantified the within- and between-family contrasts and defined a measure of family differentiation to score, on average, how well models belonging to one family distinguish from others.

We found that the baseline network family and families with increased coupling strengths or decreased amplitude (respectively, BL, HC, and LC) differentiate themselves most from other families, in both structural and functional connectivity. The differentiation score is the highest for the structural connectivity of the HC family. Moreover, we observe a relatively high resemblance between HC and LC for both structural and functional connectivity. Based on the HHG test, there is relatively strong evidence (*p* = 0.02) for distributional dependence between structural networks of HC and LC. This means that elevated coupling strength and reduced amplitude have similar effects on both the structural and the functional connectivity of adaptively rewiring oscillator models.

Altogether, perturbation to the parameters of coupled oscillators yields structural and functional differences that are fundamental for implementing cognitive functions in evolving networks. Moreover, for both the structural and the functional connectivity, perturbation leads to differentiation from the baseline. Different perturbations show specific differentiations. From a cognitive neuroscience perspective, this implies that functional and structural differentiation can be used to identify functional components in a network, in accord with the use of structural and functional connectivity measures in neuroimaging.

## CONCLUDING REMARKS

Further research may extend our findings in several ways. First, the incidental occurrence of network evolution breakdown was unexpected. Technically speaking, the matrix algebraic implementation of coupled logistic maps is sensitive to minor computational errors such as undefined division for one node (which may stem from pathological development of models), as was the case for MC2, MC3, SC1, and SC3. A different implementation could have prevented breakdown by isolating the problematic node and proceeding with the adaptive rewiring algorithm for the remaining network. However, rather than searching for an even more robust version of our algorithm, we choose to keep the current implementation for the sake of consistency with previous work, and as a warning signal that pathologies may arise as a by-product of neural network evolution. Future work may focus more specifically on such pathological development, in relation to developmental disorders in the human brain. This may require a systematic study of the probability distribution of network breakdown under a range of parametrization conditions, network sizes, and connectivity densities to find factors contributing to the pathological development of adaptively rewiring networks.

Second, a broader range of parameter variation conditions than currently imposed should be attempted. For instance, amplitude and coupling strength parameters could both deviate from baseline values simultaneously, either for the same subset of nodes or for two (overlapping or nonoverlapping) subsets. Random and patterned deviations of parameters can be studied in large-scale systems to implement perceptual and memory functions. Ultimately, the aim is to have these functions implemented in a network that simultaneously maintains its optimal structure.

This study was limited to binary, unweighted, and undirected networks. The effect of nonuniform parameters of logistic maps can be studied, via systematic search, on weighted networks with various edge weight distributions, akin to Hellrigel et al. (2019). For directed networks, see Rentzeperis, Laquitaine, and van Leeuwen (2021).

We followed the strategy to provide the simplest possible model of brain structure and function in order to avoid stacking arbitrary assumptions. Simplification is inevitable in modeling; “all models are wrong—but some are useful.” (Attributed to George Box.) For those who consider the model to pose severe limitations on the generalization of our findings to neurobiological systems, more realistic neural mass models instead of coupled maps could be a viable solution, in particular, ones that have facilities for studying the effects of **traveling waves** and spatial embedding of the network topology (Calvo Tapia, Makarov, & van Leeuwen, 2020) on network evolution, or apply phase–amplitude–frequency coupling (Chehelcheraghi, Nakatani, Steur, & van Leeuwen, 2016; Chehelcheraghi, van Leeuwen, Steur, & Nakatani, 2017) to networks of neural mass activity (Deschle, Ignacio Gossn, Tewarie, Schelter, & Daffertshofer, 2021).

Finally, despite the current limitations, our study has set an essential first step in the development of adaptively rewiring networks capable of pattern recognition and learning. Showing the principled possibility of such networks offers scope for the study of the developing, functional brain as well as for applying adaptive rewiring to sparsify artificial neural networks (Gale, Elsen, & Hooker, 2019).

## ACKNOWLEDGMENTS

Part of the research leading to this review was supported by an Odysseus grant (G.0003.12) from the Flemish Organization for Science (FWO) to Cees van Leeuwen. The computational resources and services used in this work were provided by the VSC (Flemish Supercomputer Center), funded by FWO and the Flemish Government. The authors would like to thank Ilias Rentzeperis for his feedback and comments on parts of the results.

## DATA AND CODE AVAILABILITY

The reproducible scripts used in this study are available online on the study’s repository on the Open Science Framework (https://osf.io/625d8; Haqiqatkhah & van Leeuwen, 2021). This repository also includes all model files of this study (500 files amounting to 101 GB of data, generated using VSC computational resources) and additional plots of various qualitative and quantitative network measures.

## SUPPORTING INFORMATION

Supporting information for this article is available at https://doi.org/10.1162/netn_a_00211.

## AUTHOR CONTRIBUTIONS

MohammadHossein Manuel Haqiqatkhah: Conceptualization; Data curation; Formal analysis; Investigation; Methodology; Software; Visualization; Writing – original draft. Cees van Leeuwen: Conceptualization; Funding acquisition; Investigation; Project administration; Resources; Supervision; Writing – review & editing.

## FUNDING INFORMATION

Cees van Leeuwen, Fonds Wetenschappelijk Onderzoek (https://dx.doi.org/10.13039/501100003130), Award ID: G.0003.12.

## TECHNICAL TERMS

Structural plasticity: The brain’s ability to modify its structure by means of forming and pruning neuron-to-neuron connections or changes in synaptic strength based on neural activities.
Neural mass oscillators: Simplified models of coarse-grained mesoscopic or macroscopic oscillatory activities of often large coupled populations of excitatory and inhibitory neurons and their synapses.
Chaotic attractor: An attractor (a set of values or states that a dynamic system tends to evolve toward) that is highly sensitive to the initial conditions of the system.
Poincaré section: A lower dimension in a continuous dynamic system’s state space with which the periodic orbit of the system’s flow intersects.
Limit-cycle attractors: An isolated periodic orbit or closed trajectory of a continuous dynamical system that has a two-dimensional state space.
Perceptual organization: A process that, by grouping visual elements (such as corners and edges), facilitates determining the meaning of the visual input as a whole.
Degree sequence: A sorted list of the degrees of nodes (i.e., the number of connections each node has to others) in a network.
Traveling waves: Spatially coherent neural activities that propagate across brain regions at different scales and that are hypothesized to coordinate brain circuits at longer ranges.

## Supporting Information

https://doi.org/osf.io/625d8

## References

[bib1] Avena-Koenigsberger, A., Misic, B., & Sporns, O. (2018). Communication dynamics in complex brain networks. Nature Reviews Neuroscience, 19(1), 17–33. 10.1038/nrn.2017.149, 29238085

[bib2] Berlingerio, M., Koutra, D., Eliassi-Rad, T., & Faloutsos, C. (2012). NetSimile: A scalable approach to size-independent network similarity. arXiv:1209.2684.

[bib3] Bi, G.-Q., & Poo, M.-M. (2001). Synaptic modification by correlated activity: Hebb’s postulate revisited. Annual Review of Neuroscience, 24(1), 139–166. 10.1146/annurev.neuro.24.1.139, 11283308

[bib4] Breakspear, M., Terry, J. R., & Friston, K. J. (2003). Modulation of excitatory synaptic coupling facilitates synchronization and complex dynamics in a biophysical model of neuronal dynamics. Network: Computation in Neural Systems, 14(4), 703–732. 10.1088/0954-898X_14_4_305 14653499

[bib5] Butz, M., Wörgötter, F., & van Ooyen, A. (2009). Activity-dependent structural plasticity. Brain Research Reviews, 60(2), 287–305. 10.1016/j.brainresrev.2008.12.023, 19162072

[bib6] Calvo Tapia, C., Makarov, V. A., & van Leeuwen, C. (2020). Basic principles drive self-organization of brain-like connectivity structure. Communications in Nonlinear Science and Numerical Simulation, 82, 105065. 10.1016/j.cnsns.2019.105065

[bib7] Chehelcheraghi, M., Nakatani, C., Steur, E., & van Leeuwen, C. (2016). A neural mass model of phase–amplitude coupling. Biological Cybernetics, 110(2), 171–192. 10.1007/s00422-016-0687-5, 27241189

[bib8] Chehelcheraghi, M., van Leeuwen, C., Steur, E., & Nakatani, C. (2017). A neural mass model of cross frequency coupling. PLoS ONE, 12(4), e0173776. 10.1371/journal.pone.0173776, 28380064 PMC5381784

[bib9] Clauset, A., Newman, M. E. J., & Moore, C. (2004). Finding community structure in very large networks. Physical Review E, 70(6), 066111. 10.1103/PhysRevE.70.066111, 15697438

[bib10] Costa, L. d. F., Rodrigues, F. A., Travieso, G., & Villas Boas, P. R. (2007). Characterization of complex networks: A survey of measurements. Advances in Physics, 56(1), 167–242. 10.1080/00018730601170527

[bib11] Csardi, G., & Nepusz, T. (2006). The igraph software package for complex network research. InterJournal, Complex Systems, 1695.

[bib12] Deschle, N., Ignacio Gossn, J., Tewarie, P., Schelter, B., & Daffertshofer, A. (2021). On the validity of neural mass models. Frontiers in Computational Neuroscience, 14, 581040. 10.3389/fncom.2020.581040, 33469424 PMC7814001

[bib13] Feigenbaum, M. J. (1978). Quantitative universality for a class of nonlinear transformations. Journal of Statistical Physics, 19(1), 25–52. 10.1007/BF01020332

[bib14] Gale, T., Elsen, E., & Hooker, S. (2019). The state of sparsity in deep neural networks. arXiv:1902.09574.

[bib15] Gong, P., & van Leeuwen, C. (2003). Emergence of scale-free network with chaotic units. Physica A: Statistical Mechanics and Its Applications, 321(3), 679–688. 10.1016/S0378-4371(02)01735-1

[bib16] Gong, P., & van Leeuwen, C. (2004). Evolution to a small-world network with chaotic units. EPL (Europhysics Letters), 67(2), 328. 10.1209/epl/i2003-10287-7

[bib17] Gu, Z., Eils, R., & Schlesner, M. (2016). Complex heatmaps reveal patterns and correlations in multidimensional genomic data. Bioinformatics, 32(18), 2847–2849. 10.1093/bioinformatics/btw313, 27207943

[bib18] Hahsler, M., Hornik, K., & Buchta, C. (2008). Getting things in order: An introduction to the R package seriation. Journal of Statistical Software, 25(3). 10.18637/jss.v025.i03

[bib19] Haqiqatkhah, M. H. M., & van Leeuwen, C. (2021). Adaptive rewiring on coupled logistic maps with heterogenous parameters, Open Science Framework, https://osf.io/625d8/

[bib20] Hebb, D. O. (1949). The organization of behavior (Vol. 65). New York, NY: Wiley.

[bib21] Heller, R., Heller, Y., & Gorfine, M. (2013). A consistent multivariate test of association based on ranks of distances. Biometrika, 100(2), 503–510. 10.1093/biomet/ass070

[bib22] Hellrigel, S., Jarman, N., & van Leeuwen, C. (2019). Adaptive rewiring in weighted networks. Cognitive Systems Research, 55, 205–218. 10.1016/j.cogsys.2019.02.004

[bib23] Jarman, N., Steur, E., Trengove, C., Tyukin, I. Y., & van Leeuwen, C. (2017). Self-organisation of small-world networks by adaptive rewiring in response to graph diffusion. Scientific Reports, 7(1), 13158. 10.1038/s41598-017-12589-9, 29030608 PMC5640682

[bib24] Jarman, N., Trengove, C., Steur, E., Tyukin, I., & van Leeuwen, C. (2014). Spatially constrained adaptive rewiring in cortical networks creates spatially modular small world architectures. Cognitive Neurodynamics, 8(6), 479–497. 10.1007/s11571-014-9288-y, 26396647 PMC4571644

[bib25] Kaneko, K. (1992). Overview of coupled map lattices. Chaos: An Interdisciplinary Journal of Nonlinear Science, 2(3), 279–282. 10.1063/1.165869, 12779975

[bib26] Kwok, H. F., Jurica, P., Raffone, A., & van Leeuwen, C. (2007). Robust emergence of small-world structure in networks of spiking neurons. Cognitive Neurodynamics, 1(1), 39–51. 10.1007/s11571-006-9006-5, 19003495 PMC2288955

[bib27] Meunier, D., Lambiotte, R., & Bullmore, E. T. (2010). Modular and hierarchically modular organization of brain networks. Frontiers in Neuroscience, 4. 10.3389/fnins.2010.00200, 21151783 PMC3000003

[bib28] Newman, M. E. J. (2003). Mixing patterns in networks. Physical Review E, 67(2), 026126. 10.1103/PhysRevE.67.026126, 12636767

[bib29] Newman, M. E. J. (2006). Modularity and community structure in networks. Proceedings of the National Academy of Sciences, 103(23), 8577–8582. 10.1073/pnas.0601602103, 16723398 PMC1482622

[bib30] Papadopoulos, L., Kim, J. Z., Kurths, J., & Bassett, D. S. (2017). Development of structural correlations and synchronization from adaptive rewiring in networks of Kuramoto oscillators. Chaos: An Interdisciplinary Journal of Nonlinear Science, 27(7), 073115. 10.1063/1.4994819, 28764402 PMC5552408

[bib31] R Core Team. (2019). R: A language and environment for statistical computing. Vienna, Austria.

[bib32] Rentzeperis, I., Laquitaine, S., & van Leeuwen, C. (2021). Adaptive rewiring of random neural networks generates convergent-divergent units. arXiv:2104.01418.

[bib33] Rubinov, M., Sporns, O., van Leeuwen, C., & Breakspear, M. (2009). Symbiotic relationship between brain structure and dynamics. BMC Neuroscience, 10(1), 55. 10.1186/1471-2202-10-55, 19486538 PMC2700812

[bib34] Sporns, O., & Zwi, J. D. (2004). The small world of the cerebral cortex. Neuroinformatics, 2(2), 145–162. 10.1385/NI:2:2:145, 15319512

[bib35] van den Berg, D., Gong, P., Breakspear, M., & van Leeuwen, C. (2012). Fragmentation: Loss of global coherence or breakdown of modularity in functional brain architecture? Frontiers in Systems Neuroscience, 6. 10.3389/fnsys.2012.00020, 22479239 PMC3316147

[bib36] van den Berg, D., & van Leeuwen, C. (2004). Adaptive rewiring in chaotic networks renders small-world connectivity with consistent clusters. EPL (Europhysics Letters), 65(4), 459. 10.1209/epl/i2003-10116-1

[bib37] van den Heuvel, M. P., & Sporns, O. (2011). Rich-club organization of the human connectome. Journal of Neuroscience, 31(44), 15775–15786. 10.1523/JNEUROSCI.3539-11.2011, 22049421 PMC6623027

[bib38] van Leeuwen, C., & Raffone, A. (2001). Coupled nonlinear maps as models of perceptual pattern and memory trace dynamics. Cognitive Processing, 2, 67–116.

[bib39] van Leeuwen, C., Steyvers, M., & Nooter, M. (1997). Stability and intermittency in large-scale coupled oscillator models for perceptual segmentation. Journal of Mathematical Psychology, 41(4), 319–344. 10.1006/jmps.1997.1177, 9473396

[bib40] Watts, D. J., & Strogatz, S. H. (1998). Collective dynamics of “small-world” networks. Nature, 393(6684), 440–442. 10.1038/30918, 9623998

[bib41] Wei, T., & Simko, V. (2021). R package ‘corrplot’: Visualization of a correlation matrix [Manual].

[bib42] Zhang, X., Ma, Z., Zhang, Z., Sun, Q., & Yan, J. (2018). A review of community detection algorithms based on modularity optimization. Journal of Physics: Conference Series, 1069, 012123. 10.1088/1742-6596/1069/1/012123

