# Supplementary material for: Adaptive rewiring in nonuniform coupled oscillators
Source: Netw Neurosci. 2022 Feb 1;6(1):90–117. doi: 10.1162/netn_a_00211 (PMC8959120; doi:10.1162/netn_a_00211)
Supplement: Supplementary file 1 [file netn-06-90-s001.pdf]

Haqiqatkhah, M. M. & van Leeuwen, C. (2022). Supporting information for "Adaptive rewiring in nonuniform coupled oscillators." *Network Neuroscience*, 6(1), 90–117. https://doi.org/10.1162/netn_a_00211

Supplemental Materials for "Adaptive Rewiring in Non-Uniform Coupled Oscillator"

MohamamdHossein Manuel Haqiqatkhah[1,2] and Cees van Leeuwen[1,3]
[1]KU Leuven, Belgium
[2]Utrecht University, the Netherlands
[3]TU Kaiserslautern, Germany

Derivations

Matrix-vector Realization of Coupled Logistic Maps

In the main body of the manuscript, we stated that the dynamics of a network of N coupled logistics map, each described by

$$x_{i,t+1} = (1-\varepsilon_i)(1-\alpha_i x_{it}^2) + \frac{\varepsilon_i}{|B_i|}\sum_{j\in B_i}(1-\alpha_i x_{jt}^2) \tag{S1}$$

may be re-written in matrix notation as

$$X_{t+1} = [\mathbf{1}_N - \boldsymbol{\alpha} \odot X_t \odot X_t] \odot [\mathbf{1}_N - \boldsymbol{\varepsilon} + (A_t\boldsymbol{\varepsilon}) \oslash (A_t\mathbf{1}_N)] \tag{S2}$$

The equivalence of Equations S1 and S2 can be shown as follows. First, we may write the activities of all nodes at time $t+1$ as a vector of size N, i.e.,

$$X_{t+1} = \begin{bmatrix} x_{1,t+1} \\ x_{2,t+1} \\ \vdots \\ x_{N,t+1} \end{bmatrix} = \underbrace{\begin{bmatrix} (1-\varepsilon_1)(1-\alpha_1 x_{1t}^2) \\ (1-\varepsilon_2)(1-\alpha_2 x_{2t}^2) \\ \vdots \\ (1-\varepsilon_N)(1-\alpha_N x_{Nt}^2) \end{bmatrix}}_{\mathbb{I}} + \underbrace{\begin{bmatrix} \frac{\varepsilon_1}{|B_1|}\sum_{j\in B_1}(1-\alpha_j x_{jt}^2) \\ \frac{\varepsilon_2}{|B_2|}\sum_{j\in B_2}(1-\alpha_j x_{jt}^2) \\ \vdots \\ \frac{\varepsilon_N}{|B_N|}\sum_{j\in B_N}(1-\alpha_j x_{jt}^2) \end{bmatrix}}_{\mathbb{II}}. \tag{S3}$$

We may write $\mathbb{I}$ as element-wise multiplication of two vectors, for which we use the

Hadamard operator $\odot$:

$$\begin{aligned}
\mathbb{I} &= \begin{bmatrix} 1-\varepsilon_1 \\ 1-\varepsilon_2 \\ \vdots \\ 1-\varepsilon_N \end{bmatrix} \odot \begin{bmatrix} 1-\alpha_1 x_{1t}^2 \\ 1-\alpha_2 x_{2t}^2 \\ \vdots \\ 1-\alpha_N x_{Nt}^2 \end{bmatrix} \\
&= \left(\begin{bmatrix} 1 \\ 1 \\ \vdots \\ 1 \end{bmatrix} - \begin{bmatrix} \varepsilon_1 \\ \varepsilon_2 \\ \vdots \\ \varepsilon_N \end{bmatrix} \right) \odot \left(\begin{bmatrix} 1 \\ 1 \\ \vdots \\ 1 \end{bmatrix} - \begin{bmatrix} \alpha_1 x_{1t} x_{1t} \\ \alpha_2 x_{2t} x_{2t} \\ \vdots \\ \alpha_N x_{Nt} x_{Nt} \end{bmatrix} \right) \\
&= \left(\mathbf{1}_N - \begin{bmatrix} \varepsilon_1 \\ \varepsilon_2 \\ \vdots \\ \varepsilon_N \end{bmatrix} \right) \odot \left(\mathbf{1}_N - \begin{bmatrix} \alpha_1 \\ \alpha_2 \\ \vdots \\ \alpha_N \end{bmatrix} \odot \begin{bmatrix} x_{1t} \\ x_{2t} \\ \vdots \\ x_{Nt} \end{bmatrix} \odot \begin{bmatrix} x_{1t} \\ x_{2t} \\ \vdots \\ x_{Nt} \end{bmatrix} \right) \\
&= (\mathbf{1}_N - \boldsymbol{\varepsilon}) \odot (\mathbf{1}_N - \boldsymbol{\alpha} \odot X_t \odot X_t).
\end{aligned} \tag{S4}$$

$\mathbb{III}$ can similarly be simplified using element-wise multiplication and division using Hadamard $\odot$ and $\oslash$ operators:

$$\begin{aligned}
\mathbb{III} &= \begin{bmatrix} \varepsilon_1 \\ \varepsilon_2 \\ \vdots \\ \varepsilon_N \end{bmatrix} \odot \begin{bmatrix} \sum_{j\in B_1}(1-\alpha_j x_{jt}^2) \\ \sum_{j\in B_2}(1-\alpha_j x_{jt}^2) \\ \vdots \\ \sum_{j\in B_N}(1-\alpha_j x_{jt}^2) \end{bmatrix} \odot \begin{bmatrix} \frac{1}{|B_1|} \\ \frac{1}{|B_2|} \\ \vdots \\ \frac{1}{|B_N|} \end{bmatrix} \\
&= \boldsymbol{\varepsilon} \odot \underbrace{\begin{bmatrix} \sum_{j\in B_1}(1-\alpha_j x_{jt}^2) \\ \sum_{j\in B_2}(1-\alpha_j x_{jt}^2) \\ \vdots \\ \sum_{j\in B_N}(1-\alpha_j x_{jt}^2) \end{bmatrix}}_{\mathbb{IIII}} \oslash \underbrace{\begin{bmatrix} |B_1| \\ |B_2| \\ \vdots \\ |B_N| \end{bmatrix}}_{\mathbb{IV}}.
\end{aligned} \tag{S5}$$

$\mathbb{IIII}$ is sum of activities of nodes connected to node i, and we may calculate $\mathbb{IIII}$ by left-multiplying the adjacency matrix A_t by the vector of activities of all nodes:

$$\mathbb{IIII} = A_t(\mathbf{1}_N - \boldsymbol{\alpha} \odot X_t \odot X_t). \tag{S6}$$

This multiplication implies that $\mathbb{IIII}_i$ (the ith element of the vector) is equal to $\sum_j a_{ij}(1 - \alpha_i x_{it}^2)$, in which a_{ij} is the element on row i and column j of A_t, which is 1 if nodes i and j are connected and 0 otherwise. In other words, this multiplication serves the selection of neighboring nodes and summation of their values.

In a similar manner, $\mathbb{IV}$ is a vector in which the ith element is the number of nodes connected to node i. Thus, this vector is equal to row-wise summation of A_t, which can be calculated using matrix multiplication via

$$\mathbb{IV} = A_t \mathbf{1}_N. \tag{S7}$$

Thus, given commutative, associative, and distributive properties of Hadamard operators, we have

$$\begin{aligned}\mathbb{III} &= \boldsymbol{\varepsilon} \odot \left[A_t(\mathbf{1}_N - \boldsymbol{\alpha} \odot X_t \odot X_t)\right] \oslash (A_t\mathbf{1}_N) \\ &= (\boldsymbol{\varepsilon} A_t) \odot (\mathbf{1}_N - \boldsymbol{\alpha} \odot X_t \odot X_t) \oslash (A_t\mathbf{1}_N) \\ &= (\mathbf{1}_N - \boldsymbol{\alpha} \odot X_t \odot X_t) \odot (\boldsymbol{\varepsilon} A_t) \oslash (A_t\mathbf{1}_N).\end{aligned} \tag{S8}$$

Putting Equations S4 and S8 together and rearranging terms proves the equivalence:

$$\begin{aligned}X_{t+1} =& \mathbb{I} + \mathbb{III} \\ =& (\mathbf{1}_N - \boldsymbol{\varepsilon}) \odot (\mathbf{1}_N - \boldsymbol{\alpha} \odot X_t \odot X_t) \\ &+ (\mathbf{1}_N - \boldsymbol{\alpha} \odot X_t \odot X_t) \odot (\boldsymbol{\varepsilon} A_t) \oslash (A_t\mathbf{1}_N) \\ =& (\mathbf{1}_N - \boldsymbol{\alpha} \odot X_t \odot X_t) \odot \left[(\mathbf{1}_N - \boldsymbol{\varepsilon}) + (\boldsymbol{\varepsilon} A_t) \oslash (A_t\mathbf{1}_N)\right].\end{aligned} \tag{S9}$$

Methods

Networks as Distributions

Network statistics can describe network structures either locally or globally. Local measures are suitable for node-wise (or clique-wise) comparisons, while the global measures are aggregates of some local properties that provide summary statistics for the structure. Whereas local measures hardly lead to a holistic description of networks (as the nodes are usually described in isolation from each node) in their aggregation for deriving global measures, structural information is sacrificed. Therefore, neither local nor global measures are optimally suitable for the comparison of networks. A possible solution is to use the distribution of several local measures for network comparison.

Berlingerio et al. (2012) suggest characterizing each node i of the network with a seven-dimensional feature vector consisting of the following local measures that capture characteristics of the node and its first- and second-order neighbors:

1. $d_i = |N(i)|$, the number of neighbors, or degree ($N(i)$ is the set of first-order neighbors of node i, i.e., the nodes directly connected to i);
2. c_i, local clustering coefficient, which is the number of triangles connected to node i over the number of connected triples centered on node i;
3. $\overline{d_{N(i)}} = \frac{1}{d_i} \sum_{\forall j \in N(i)} d_j$, the average degree of node's neighbors;
4. $\overline{c_{N(i)}} = \frac{1}{d_i} \sum_{\forall j \in N(i)} c_j$, the average clustering coefficient of the node's neighbors;
5. $|E_{ego(i)}|$, the number of edges in the *egonet* of node i; the egonet of node i, referred to as $ego(i)$, is the subset of the network including node i, its first-order neighbors ($N(i)$), and the edges among $N(i)$;
6. $|E^{\circ}{}_{ego(i)}|$, the number of outgoing edges from the egonet of node i; and
7. $|N(ego(i))|$, the number of neighbors of the egonet of node i.

Although more local features could be added to this vector, Berlingerio and colleagues have shown that these features suffice in practice for comparison of networks.

The NetSimile Method

The distribution of local features enables the comparison of their summary statistics. In NetSimile (Berlingerio et al., 2012), the feature distribution (which is a $nodes \times features$ matrix) is aggregated into a 35-dimensional "signature vector" consisting of five summary statistics for each feature: median, mean, standard deviation, skewness, and kurtosis. The comparison of networks is thus reduced to calculating distances (or similarities) of the signature vectors. NetSimile is superior to other methods of inferring network similarity, as its computational complexity grows linearly with network size. More importantly, NetSimile allows the comparison of networks of different sizes.

Ranks and values of summary statistics characterize the overall shape of distributions and thus are highly diagnostic for their comparison (Berlingerio et al., 2012). Hence, the signature vectors are akin to ranked lists. It has been shown that the Canberra distance, defined in Equation S10 for two vectors V and W of size n, is an appropriate measure of dissimilarity for ranked lists (Jurman et al., 2009), as it is sensitive to small distances from zero and normalizes the pairwise distances of features by their absolute values. Moreover, Berlingerio et al. (2012) report that Canberra distance outperforms other distance measures in discriminating signature vectors, a desirable property of a dissimilarity measure for the task at hand.

$$\mathrm{Ca}(V, W) = \sum_{\substack{i=1 \\ |v_i|+|w_i|\neq 0}}^{n} \frac{|v_i - w_i|}{|v_i| + |w_i|}. \tag{S10}$$

We use this dissimilarity metric in the pairwise comparison of the signature vectors derived from the NetSimile algorithm. However, NetSimile does not allow hypothesis testing to infer significance levels for the distances. Berlingerio et al. (2012) suggest hypothesis testing for the independence of the distributions by pairwise comparison of the univariate distributions of the features and aggregating their p-values through averaging or choosing the maximum values. The authors report that neither Mann–Whitney nor Kolmogorov–Smirnov tests—which are nonparametric tests without any assumption for the distributions being compared—yield meaningful discrimination among networks. Notably, their approach of hypothesis testing ignores the multivariate dependencies amongst the features. Hence, we use another method to test the independence of distributions which is discussed below.

Hypothesis Testing for Similarities of Network Distributions

The significance tests used by Berlingerio et al. (2012) posit multivariate independence among features and lacks what the authors call "discrimination power." To tackle this issue, one must use multivariate dependence tests. Since parametric dependence tests make assumptions about the distributions being compared, we used the Heller–Heller–Gorfine (HHG) nonparametric permutation test of multivariate dependence (Heller et al., 2013) implemented in the *HHG* R package (Brill et al., 2018). HHG is a consistent omnibus test for the null hypothesis of distributional independence, i.e., that the joint distribution of two multivariate random variables X and Y is equal to the multiplication of the marginal

distributions of those variables. Equation S11 shows the null and alternative hypotheses:

$$\begin{cases} H_0: & F_{XY}(x,y) = F_X(x)F_Y(y) \\ H_1: & F_{XY}(x,y) \neq F_X(x)F_Y(y) \end{cases}, \quad \forall x,y. \tag{S11}$$

HHG has a reasonable computational complexity and uses norm distance matrices of the samples taken from X and Y separately. The technical details of this method are beyond the scope of this paper. In short, HHG iteratively forms hyperspheres in the joint space of $F_{XY}(x,y)$, and based on the implications of the null hypothesis, quantifies evidence against H_0 by likelihood ratio or Pearson's Chi-square tests statistics over contingency tables. From these tests, one can drive permutation p-values that can be interpreted as evidence against the null hypothesis of independence of the distributions. Hence, the lower the p-value, the more evidence favoring the dependence of the distributions being compared. Loosely speaking, one can treat the p-values derived from HHG methods as a form of distributional dissimilarity; the lower the p-value, the more "similar" the distributions are to each other. This interpretation allows us to compare non-significant p-values as relative measures of resemblance.

The *hhg.test()* function in *HHG* package runs the test for a number of permutations on distance matrices of the samples in X and Y. It outputs four different permutation p-values based on sums or maximum values of likelihood ratio or Chi-square test scores of all 2×2 contingency tables. We let HHG run for 2000 permutations for each pairwise comparison and extracted permutation p-value for the maximum of likelihood ratio score statistics as it yielded higher discriminative power compared to other test statistics.

References

Berlingerio, M., Koutra, D., Eliassi-Rad, T., & Faloutsos, C. (2012). NetSimile: A scalable approach to size-independent network similarity. *arXiv:1209.2684 [physics, stat]*.

Breakspear, M., Terry, J. R., & Friston, K. J. (2003). Modulation of excitatory synaptic coupling facilitates synchronization and complex dynamics in a biophysical model of neuronal dynamics. *Network: Computation in Neural Systems*, *14*(4), 703–732. https://doi.org/10.1088/0954-898X_14_4_305

Brill, B., Heller, Y., & Heller, R. (2018). Nonparametric independence tests and k-sample tests for large sample sizes using package HHG. *The R Journal*, *10*(1), 424. https://doi.org/10.32614/RJ-2018-008

Heller, R., Heller, Y., & Gorfine, M. (2013). A consistent multivariate test of association based on ranks of distances. *Biometrika*, *100*(2), 503–510. https://doi.org/10.1093/biomet/ass070

Jurman, G., Riccadonna, S., Visintainer, R., & Furlanello, C. (2009). Canberra distance on ranked lists. *Proceedings of Advances in Ranking NIPS 09 Workshop*, 22–27.

Rubinov, M., Sporns, O., van Leeuwen, C., & Breakspear, M. (2009). Symbiotic relationship between brain structure and dynamics. *BMC Neuroscience*, *10*(1), 55. https://doi.org/10.1186/1471-2202-10-55

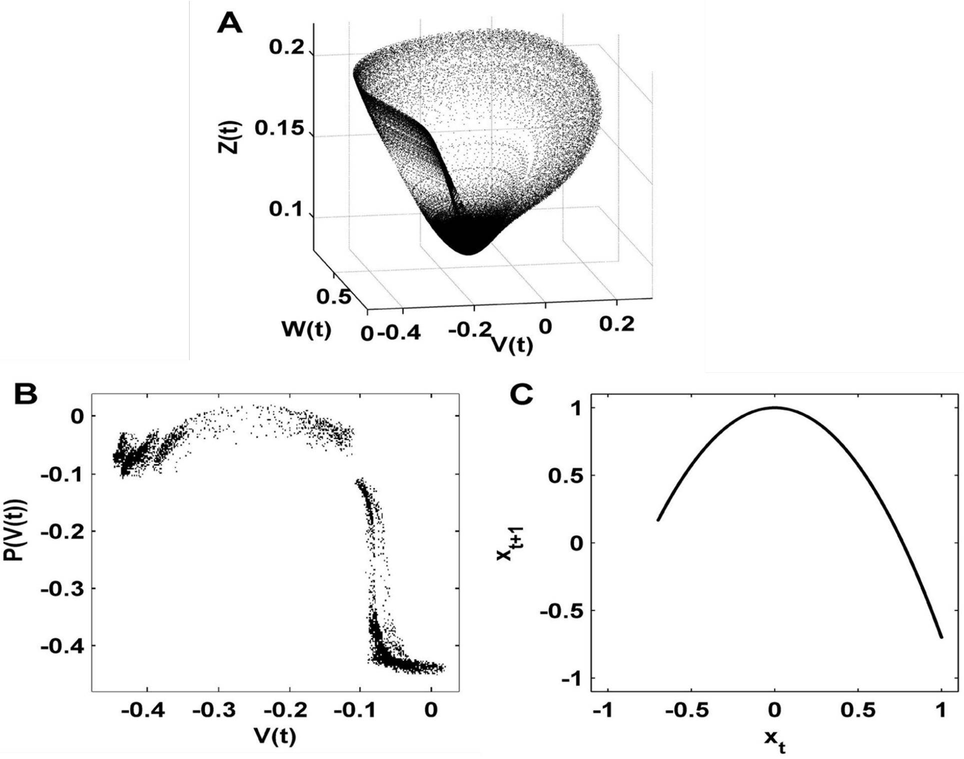

Figure S1

Dimension reduction of nonlinear neuronal dynamics. (A) Phase space attractor of a three-dimensional neural mass flow. This attractor is an illustration of the dynamics generated by the flow of a neural mass mode (cf. Breakspear et al., 2003). The dynamical variables represent the mean membrane potential of pyramidal (V) and inhibitory (Z) neurons, and the average number of open potassium ion channels (W). (B) Poincaré first return map from the same attractor (cf. Breakspear et al., 2003); this map captures key features of the neural mass flow, by following each trajectory from one intersection (V) of the attractor to the next (P(V)). (C) The quadratic logistic map that has the same unimodal topology as the neural mass Poincaré return map. While the logistic map lacks the "thickness" of the neural mass map, it is several orders of magnitude faster to compute, hence allowing more detailed quantitative analysis. Figure and caption adapted from "Symbiotic relationship between brain structure and dynamics," by. M. Rubinov, O. Sporns, C. van Leeuwen, and M. Breakspear, 2009, BMC Neuroscience, 10*(1), 55. CC BY 2.0.*

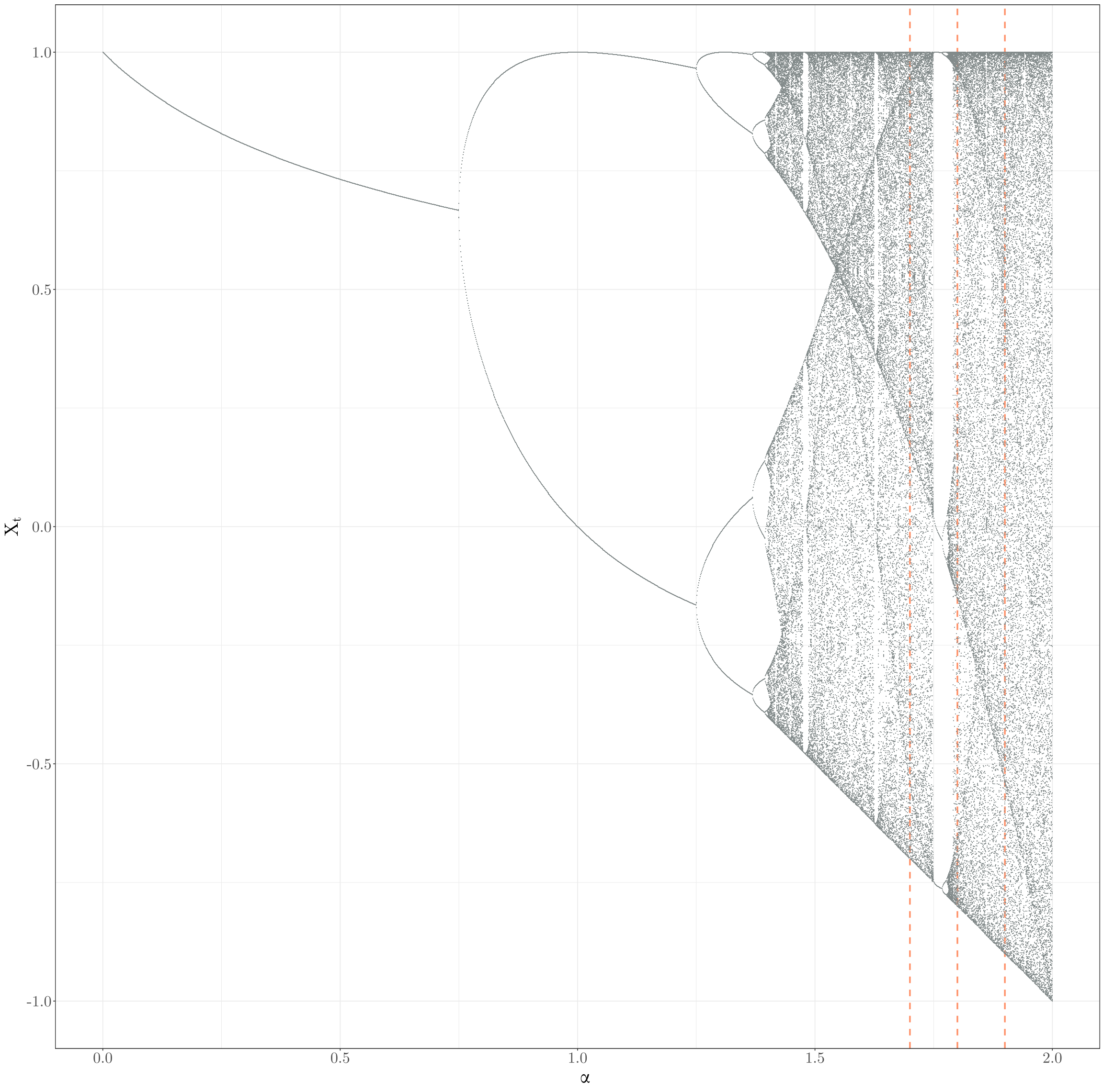

Figure S2

Feigenbaum diagram of the values of the 200 draws of logistic maps (after a burn-in period of 4000 iterations). For three levels of amplitude used in this study (i.e., $\alpha = 1.7, 1.8, or 1.9$*; vertical lines), logistic maps exhibit chaotic behavior, making them suitable dynamic elements in our models.*
